# Geldanamycin Enhances Retrograde Transport of Shiga Toxin in HEp-2 Cells

**DOI:** 10.1371/journal.pone.0129214

**Published:** 2015-05-27

**Authors:** Anne Berit Dyve Lingelem, Ieva Ailte Hjelseth, Roger Simm, Maria Lyngaas Torgersen, Kirsten Sandvig

**Affiliations:** 1 Centre for Cancer Biomedicine, Faculty of Medicine, University of Oslo, Oslo, Norway; 2 Department of Molecular Cell Biology, Institute for Cancer Research, Oslo University Hospital, Montebello, Oslo, Norway; 3 Department of Biosciences, University of Oslo, Oslo, Norway; Cambridge University, UNITED KINGDOM

## Abstract

The heat shock protein 90 (Hsp90) inhibitor geldanamycin (GA) has been shown to alter endosomal sorting, diverting cargo destined for the recycling pathway into the lysosomal pathway. Here we investigated whether GA also affects the sorting of cargo into the retrograde pathway from endosomes to the Golgi apparatus. As a model cargo we used the bacterial toxin Shiga toxin, which exploits the retrograde pathway as an entry route to the cytosol. Indeed, GA treatment of HEp-2 cells strongly increased the Shiga toxin transport to the Golgi apparatus. The enhanced Golgi transport was not due to increased endocytic uptake of the toxin or perturbed recycling, suggesting that GA selectively enhances endosomal sorting into the retrograde pathway. Moreover, GA activated p38 and both inhibitors of p38 or its substrate MK2 partially counteracted the GA-induced increase in Shiga toxin transport. Thus, our data suggest that GA-induced p38 and MK2 activation participate in the increased Shiga toxin transport to the Golgi apparatus.

## Introduction

The benzoquinoid ansamycin antibiotic geldanamycin (GA) produced by *Streptomyces hygroscopicus* is a potent inhibitor of Hsp90 proteins, and has been extensively studied due to its anti-tumor activity [1,2]. Hsp90 proteins are ubiquitously and abundantly expressed molecular chaperones whose main function is to stabilize proteins and assist in protein folding. The cytosolic Hsp90 has been best characterized, but other compartment-specific Hsp90 proteins also exist [2–4]. More than 200 client proteins of Hsp90 have so far been identified, many of which are oncoproteins [3,4]. Hsp90 is also upregulated in many cancer types and inhibition of Hsp90 affects multiple oncogenic pathways simultaneously, making Hsp90 an attractive target for cancer treatment [2,5]. GA binds to the ATP binding pocket of Hsp90, thereby interrupting its chaperone cycle, leading to degradation of many of the client proteins [1,2].

Upon GA treatment, the Hsp90 client protein ErbB2 is internalized and sorted into the lysosomal pathway for degradation [6,7]. The lysosomal targeting was recently suggested to be caused by GA-induced morphological changes of endosomal compartments [7]. Importantly, GA treatment induced missorting of the transferrin receptor, which is a commonly used marker for the recycling pathway, to multivesicular bodies [7]. Thus, GA seems to have some impact on the normal endosomal sorting process. In endosomes, cargo is not only sorted into the lysosomal and recycling pathways; it can also be selected for retrograde transport to the Golgi apparatus. The retrograde pathway is important for the retrieval of Golgi- and ER-resident receptors involved in secretion, as well as for the bulk retrieval of membrane lipids to maintain organelle integrity. Several protein toxins, such as Shiga toxin, ricin, cholera toxin and pertussis exotoxin, exploit the retrograde pathway to reach their intracellular target and to avoid lysosomal degradation ([8–10] and references therein).

In this study, we have investigated whether GA affects the sorting of cargo into the retrograde pathway using Shiga toxin as a model protein. Shiga toxins are bacterial protein toxins produced by *Shigella dysenteriae* and enterohemorrhagic *Escherichia coli* (reviewed in [9]). Shiga toxin consists of a toxic A-moiety connected to a non-toxic B-pentamer which is responsible for binding to the toxin receptor globotriaosylceramide (Gb3) on the cell surface. After internalization, the toxin is transported from endosomes via the *trans*-Golgi network (TGN) and Golgi apparatus to the ER, from where the enzymatically active part is translocated to the cytosol and inhibits protein synthesis. We here show that GA treatment strongly enhances the transport of Shiga toxin to the Golgi without a concomitant increase in endocytic uptake of the toxin or perturbed recycling, suggesting a specific effect on the endosome-to-Golgi transport step. Moreover, GA was found to activate p38, and inhibitors of p38 or its substrate MK2 counteracted the GA-induced increase in Shiga toxin transport to the Golgi apparatus, indicating that upon GA treatment, activation of the p38 pathway contributes positively to retrograde transport.

## Results

### GA enhances retrograde transport of Shiga toxin to the Golgi apparatus

**Fig 1:**
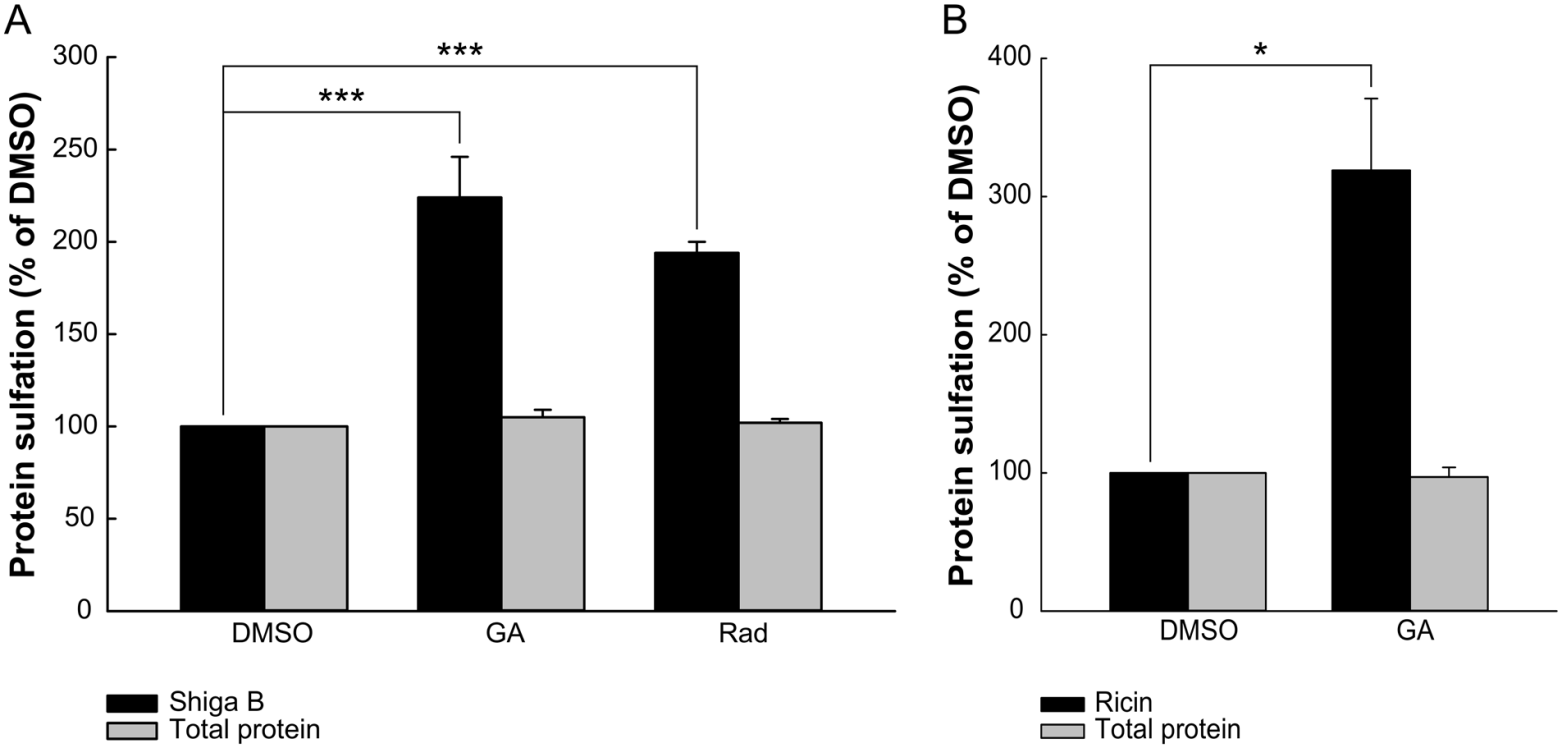
Retrograde transport of Shiga toxin and ricin is increased upon Hsp90 inhibition. HEp-2 cells were treated with 10 μM GA or 1 μM radicicol (Rad) for 30 min before 2 μg/ml Shiga B-sulf2 (A) or 4 μg/ml ricin sulf-1 (B) was added and the incubation continued for 1.5 h. The amount of sulfated toxin and the total protein sulfation was determined as described in Materials and Methods. The toxin sulfation (black bars) and total protein sulfation (grey bars) are expressed relative to control treatment (DMSO) and are plotted as mean values + SEM, *n* ≥ 3. * p ≤ 0.05, *** p ≤ 0.005, paired Student’s *t*-test.

As GA has previously been shown to alter endosomal sorting and endosome morphology, we wanted to investigate if this drug also affects the sorting of cargo into the retrograde transport pathway. To this end, we used Shiga toxin as a model protein, and to measure toxin transport to the TGN, we take advantage of the sulfation process that is mediated by TGN-localized sulfotransferases [11]. By using modified protein toxins containing sulfation sites, we can detect toxins that have reached the TGN, by measuring the association of radioactive sulfate with these toxins. We found that GA treatment more than doubled the sulfation of the modified B-subunit of Shiga toxin, Shiga B-sulf2, in HEp-2 cells (Fig 1A), indicating increased transport to the TGN. GA treatment did not alter the sulfation process per se, as total protein sulfation was unaffected (Fig 1A). To corroborate that the GA-induced increase in Shiga toxin transport is mediated by Hsp90 inhibition, we tested the structurally different Hsp90 inhibitor radicicol, which also binds to the ATP-binding pocket of Hsp90. Treatment with radicicol gave a similar increase in Shiga toxin sulfation (Fig 1A). Also, the second generation synthetic drug NVP-AUY922 gave a significant, albeit smaller, increase in Shiga toxin sulfation (S1 Fig). Together, this indicates that Hsp90, either directly or indirectly, is involved in the regulation of Shiga toxin retrograde transport.

Although some components are known to be common regulators for trafficking in the retrograde direction, it has been shown that certain cargo molecules also have distinct requirements for their retrograde transport (reviewed in [12,13]). To examine whether GA-treatment alters retrograde transport in general or Shiga toxin transport in particular, we measured the TGN transport of the plant toxin ricin. Ricin follows a similar pathway as Shiga toxin, although it binds to different receptors and its transport is somewhat differently regulated [10,14–21]. However, as for Shiga toxin, treatment with GA potently increased ricin sulfation (Fig 1B), showing that the effect of GA is not limited to retrograde transport of Shiga toxin.

**Fig 2:**
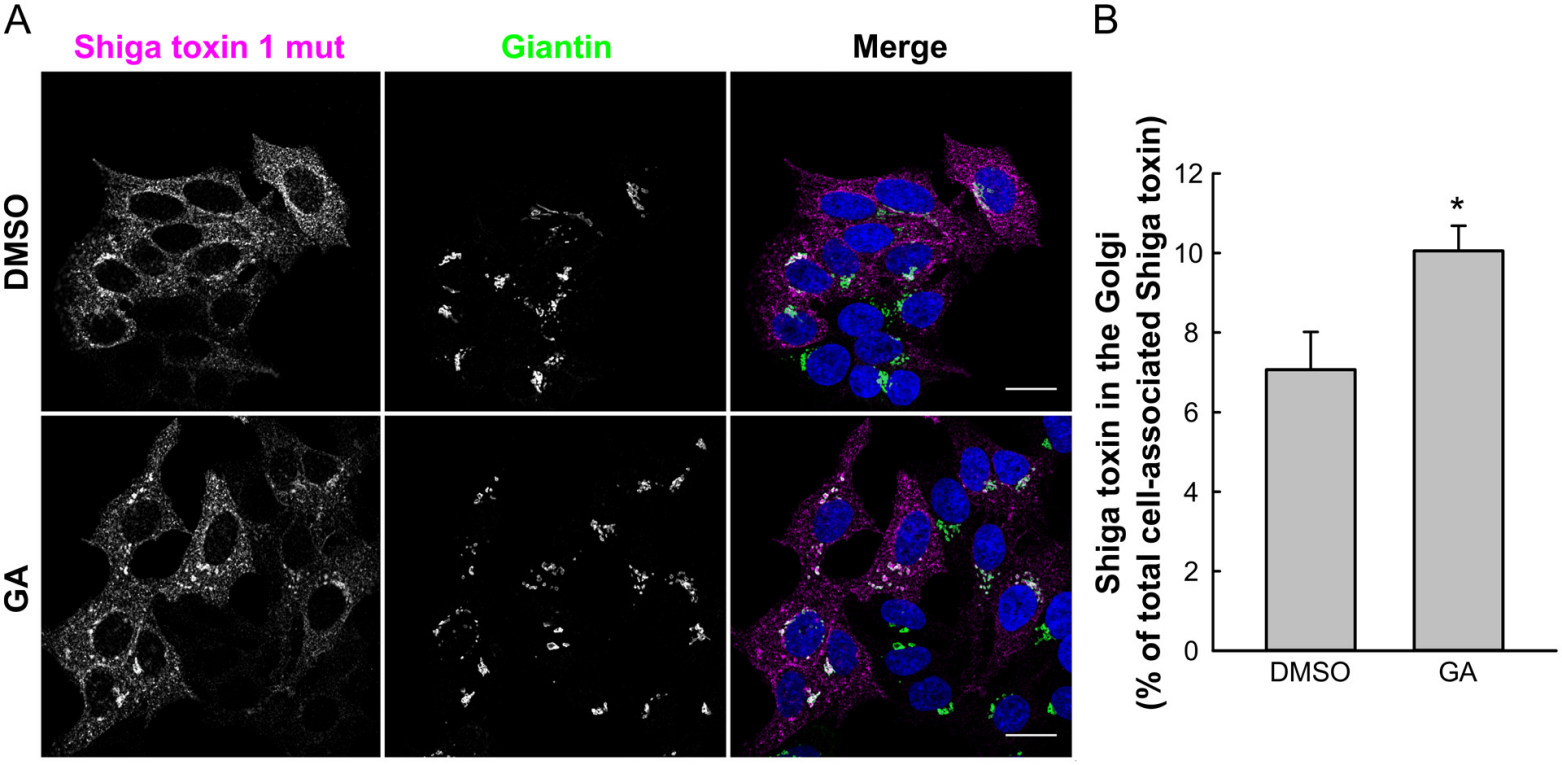
GA enhances Shiga toxin localization to the Golgi. (A) HEp-2 cells were treated with 10 μM GA for 30 min before ~100 ng/ml Shiga toxin 1 mutant was added and the incubation continued for 30 min. Subsequently, cells were fixed, permeabilized and stained with antibodies against Shiga toxin (magenta) and giantin (green). DAPI is shown in blue. Scale bar 20 μm. (B) The amount of Shiga toxin 1 mutant that has reached the Golgi was quantified as the Shiga toxin 1 mutant intensity in giantin-positive structures relative to the intensity of total cell-associated Shiga toxin 1 mutant in individual cells using Fiji software and plotted as mean values + SEM. *n* = 4, with at least 65 cells quantified for each condition. * p ≤0.05, paired Student’s *t*-test.

The GA-induced increase in Shiga toxin transport was verified by immunofluoresence confocal microscopy. Although Shiga toxin showed a variable overlap with the Golgi marker giantin in both control and GA-treated cells (Fig 2A), in total, more toxin was present in giantin-positive structures in GA-treated cells (Fig 2B), which is in agreement with the sulfation data.

**Fig 3:**
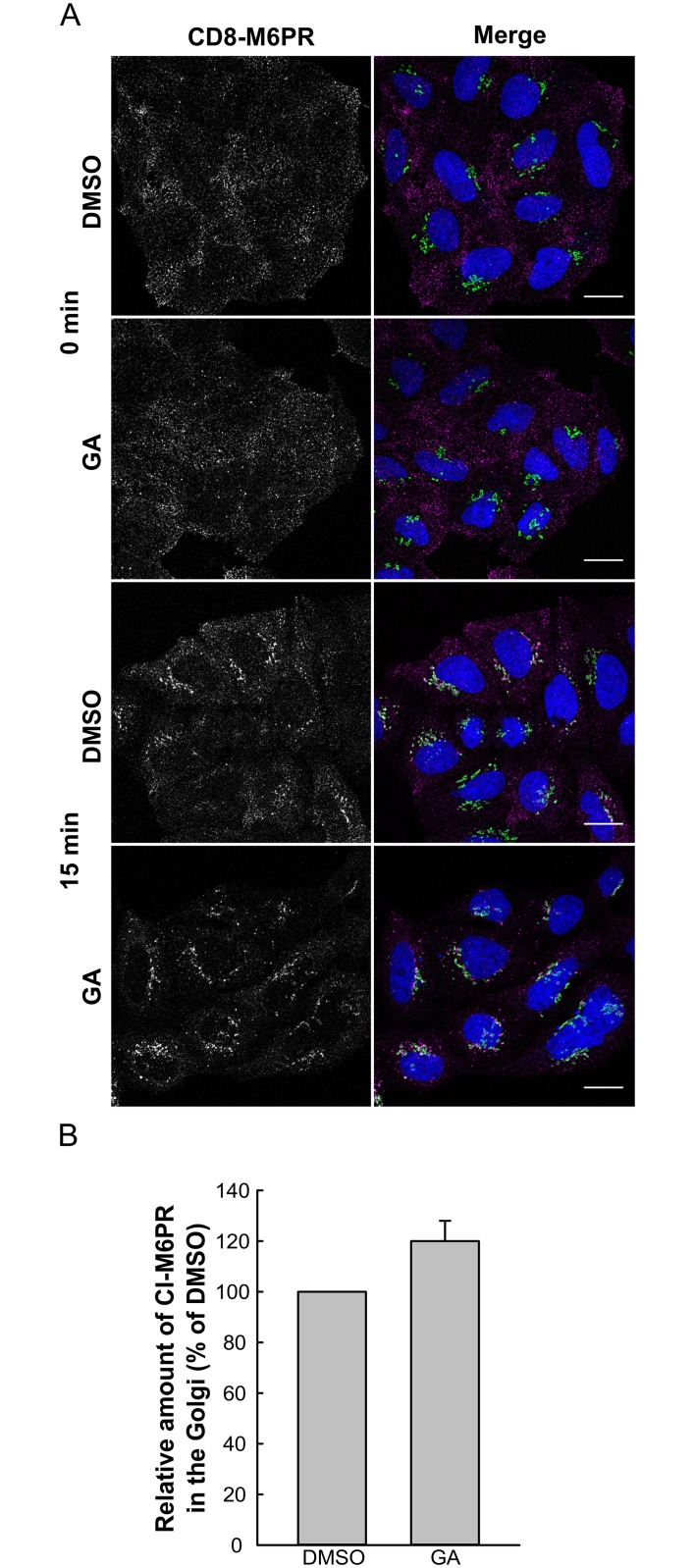
GA enhances CI-M6PR localization to the Golgi. (A) HeLa cells stably expressing CD8-M6PR fusion protein were treated with 10 μM GA for 30 min at 37°C before they were chilled and incubated with 10 μg/ml CD8 antibody at 4°C for 30 min. The CD8 antibody chase was performed for 0 or 15 min at 37°C. Subsequently, cells were fixed, permeabilized and stained with antibodies against CD8 (magenta) and giantin (green). DAPI is shown in blue. Scale bar 20 μm. (B) The amount of CI-M6PR that has reached the Golgi was quantified as the CI-M6PR intensity in giantin-positive structures relative to the intensity of total cell-associated CI-M6PR in individual cells using Fiji software. The data was normalized to control samples (DMSO) in individual experiments and plotted as mean values + SEM. *n* = 3, with at least 30–50 cells quantified for each condition.

To further investigate the ability of GA to regulate retrograde transport, we studied retrieval of the cation-independent mannose 6-phosphate receptor (CI-M6PR), which carries newly synthesized lysosomal enzymes from the Golgi apparatus to endosomes. After cargo release, the CI-M6PR is transported back to the Golgi for additional rounds of cargo transport [12]. A proportion of the CI-M6PR is transiently localized to the plasma membrane before being rapidly internalized, and this can be exploited to study its retrograde transport [22]. CI-M6PR transport was investigated by immunofluorescence confocal microscopy using a HeLa cell line stably expressing the CD8-M6PR fusion protein [23]. CD8-M6PR present at the cell surface was labeled with an antibody against CD8 and chased into the cells for 0 or 15 min. Although our data did not reach statistical significance, we see a tendency of increased CI-M6PR transport to the Golgi apparatus in GA-treated cells (Fig 3).

### GA does not increase the endocytic uptake of Shiga toxin

**Fig 4:**
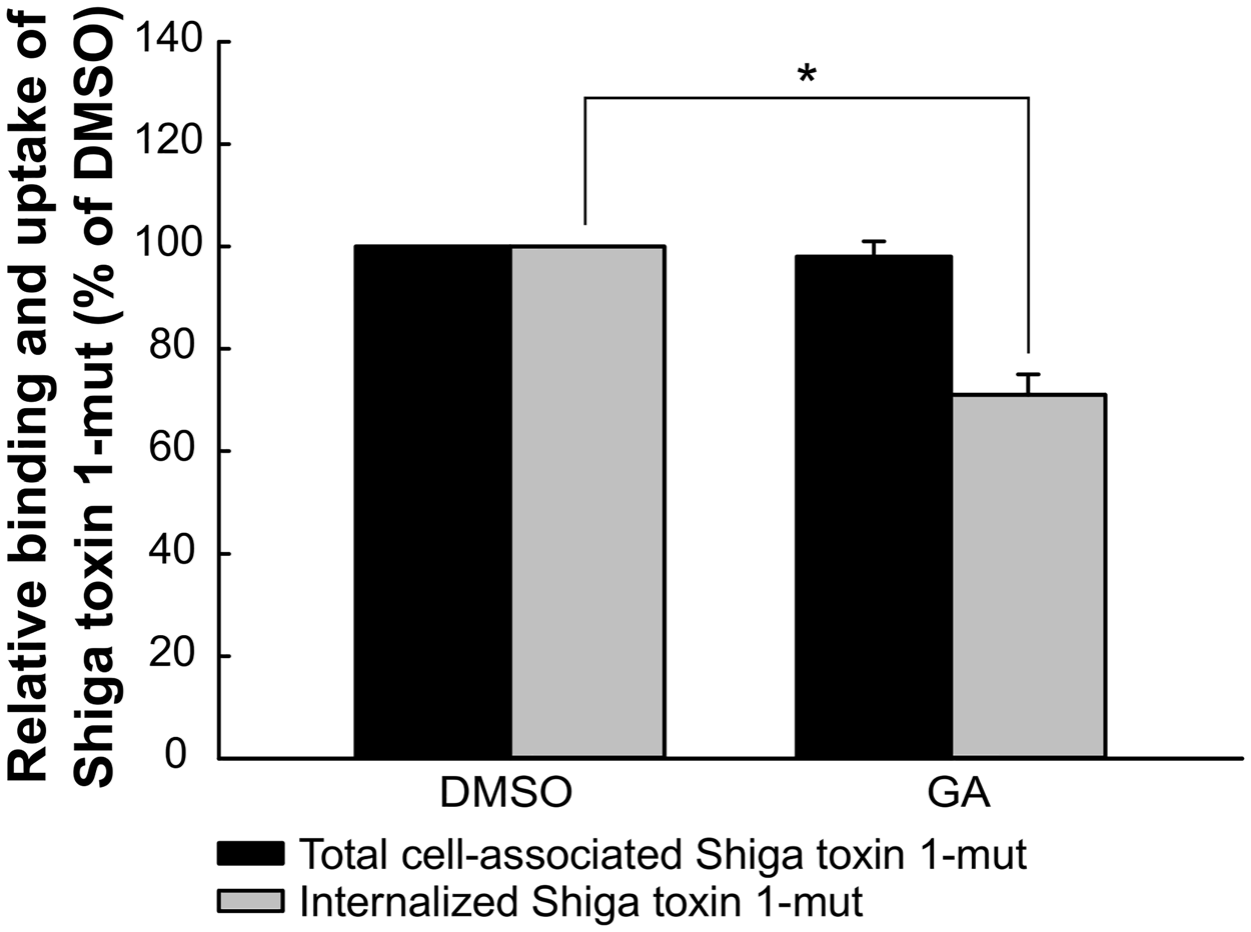
Shiga toxin endocytosis is not increased by GA treatment. HEp-2 cells were preincubated with 10 μM GA for 30 min at 37°C and subsequently incubated with 40 ng/ml biotinylated Shiga toxin 1 mutant for 20 min. The amount of internalized or total cell-associated toxin was quantified as described in Materials and Methods. Mean values + SEM of total cell-associated (black bars) and internalized (grey bars) Shiga toxin 1 mutant are presented as percentage of control (DMSO), *n* = 3. * p ≤ 0.05 paired Student’s *t*-test.

Inhibition of Hsp90 activity leads to internalization and subsequent degradation of several receptors. To determine if the enhanced retrograde transport of Shiga toxin was caused by increased internalization, we measured the endocytic uptake of Shiga toxin upon GA treatment. The total amount of cell-associated toxin was not altered by GA treatment, but there was a slight decrease in the internalization of Shiga toxin (Fig 4). Clearly, the increased retrograde transport of Shiga toxin is not due to increased internalization. GA had no effect on ricin endocytosis (S2 Fig).

### GA does not alter Shiga toxin recycling

**Fig 5:**
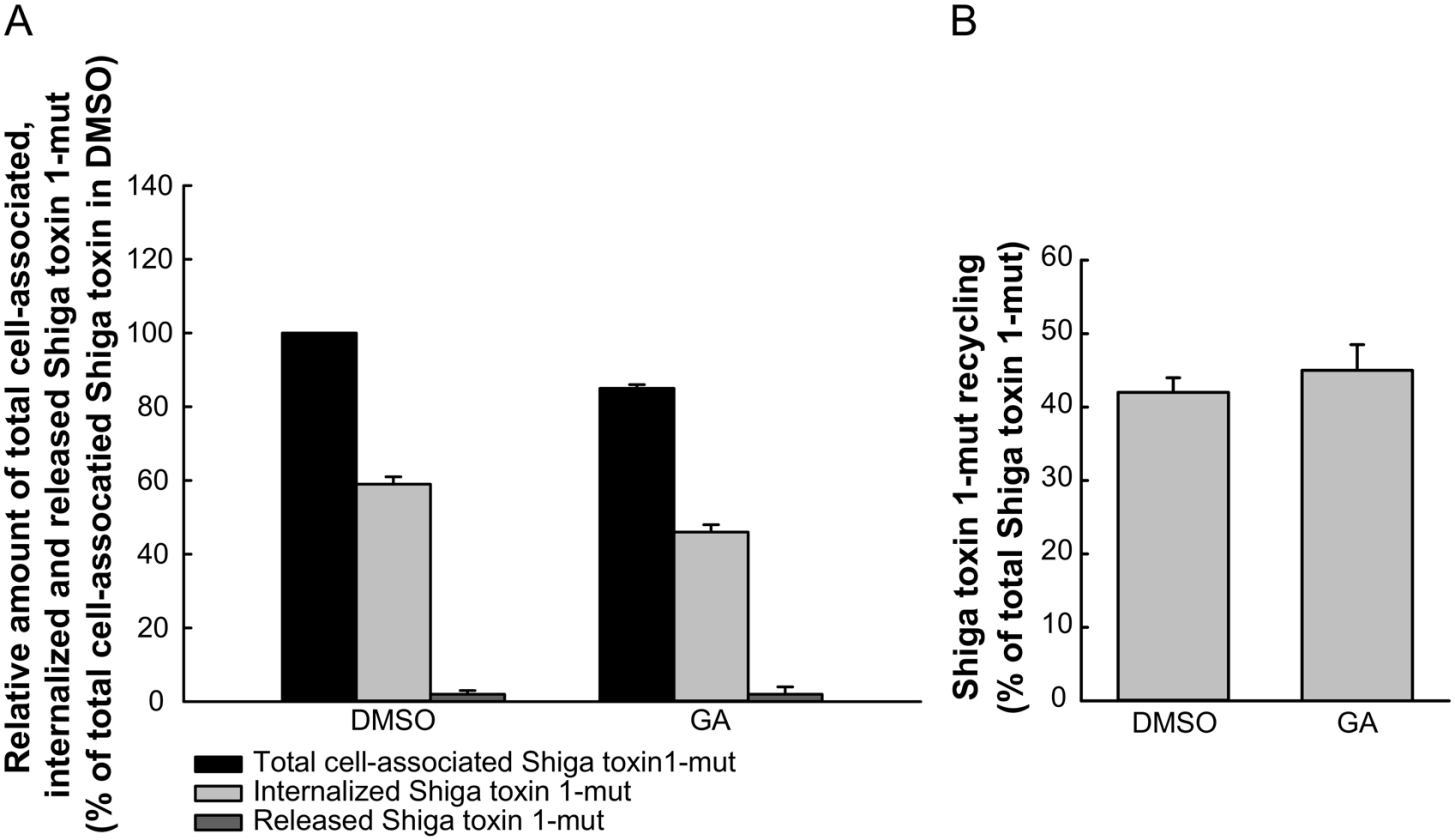
GA treatment does not alter Shiga toxin recycling. HEp-2 cells were preincubated with 10 μM GA for 30 min at 37°C and subsequently incubated with 40 ng/ml biotinylated Shiga toxin 1 mutant for 30 min. Signals from cell surface-associated Shiga toxin were removed and the toxin chased for another 30 min in the presence of inhibitor. The amount of internalized, total cell-associated toxin and toxin released to the medium was quantified as described in Materials and Methods. (A) Mean values + SD of total cell-associated (black bars), internalized (light grey bars) and released (dark grey bars) Shiga toxin 1 mutant are presented as percentage of control (DMSO). (B) Shiga toxin recycling was calculated as described in Materials and Methods and is presented as mean values + SD, *n* = 2.

GA treatment has been shown to increase degradation of the transferrin receptor by redirecting its transport from the recycling pathway into the degradative pathway [7]. Thus, the increased retrograde transport of Shiga toxin could possibly have been a consequence of perturbed recycling. So far, Shiga toxin recycling has not been extensively studied and there is no established method for its measurement. Determination of Shiga toxin recycling is complicated by the fact that once bound, Shiga toxin is strongly associated with its receptor Gb3 [24] and is unlikely to dissociate from Gb3 after recycling. To measure Shiga toxin recycling, we have modified the method used to determine Shiga toxin endocytosis, which allows us to distinguish between cell surface-bound and internalized toxin. The amount of internalized, cell-associated, and released Shiga toxin was determined as described in detail in Materials and Methods. As shown in Fig 5A, a large fraction of the internalized Shiga toxin is indeed recycled to the cell surface, but only a small fraction is released to the medium. The total amount of cell-associated Shiga toxin is slightly decreased in GA-treated cells, which is in agreement with the reduction in Shiga toxin internalization shown above, however, the fraction of Shiga toxin being recycled is not altered by GA treatment (Fig 5B). Similarly, neither ricin recycling nor degradation was affected by GA treatment (S3 Fig). Thus, it seems like the increase in retrograde transport of these toxins after GA treatment is not caused by a change of transport in other pathways.

### GA activates p38 which contributes to increased retrograde transport

**Fig 6:**
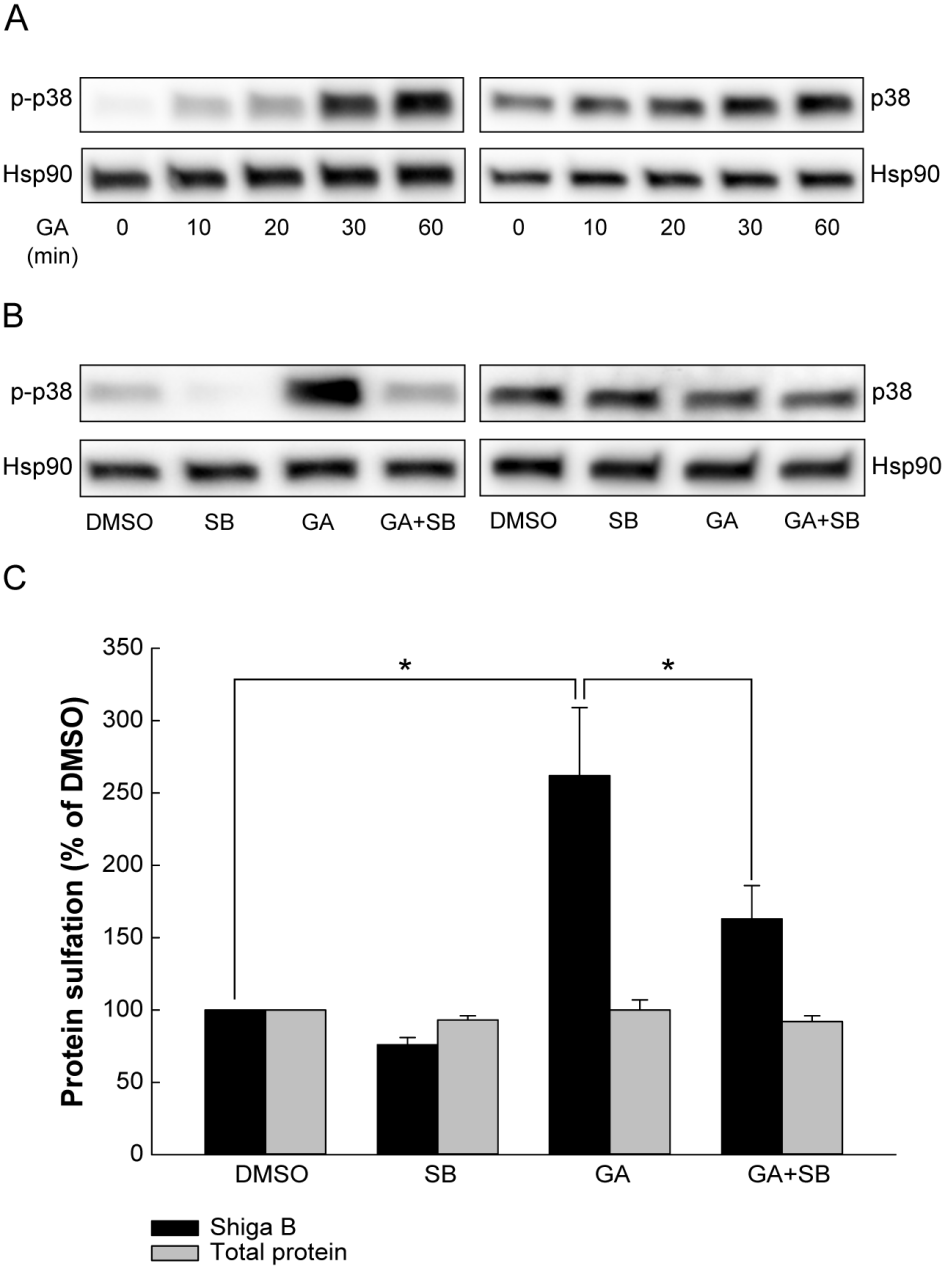
GA activates p38 which contributes to retrograde transport. HEp-2 cells were serum-starved in HEPES-buffered medium before incubation with (A) 10 μM GA for the indicated time points or with (B) 10 μM of the indicated inhibitors for 30 min. The cells were lysed and proteins were separated by SDS-PAGE. Blots were probed with the indicated antibodies. Hsp90 was used as a loading control. (C) Cells were preincubated with 10 μM GA in combination with 10 μM SB 203580 (SB) for 30 min and subsequently incubated with Shiga B-sulf2 for 1 h. The Shiga B sulfation (black bars) and total protein sulfation (grey bars) are expressed relative to control treatment (DMSO) and are plotted as mean values + SEM, *n* = 4. * p ≤ 0.05, paired Student’s *t*-test.

As Hsp90 inhibition by GA affects a vast number of proteins, GA or Hsp90 are not necessarily directly involved in the alterations of endosomal sorting. GA has been reported to induce autophosphorylation and activation of the mitogen activated protein kinase p38 by preventing its association with the Hsp90-Cdc37 complex [25]. This is of particular interest with respect to toxin transport as it has previously been shown that p38 activity is important for retrograde transport of Shiga toxin to the Golgi apparatus [18,26]. We were therefore interested to see whether p38 activity would contribute to the GA-induced increase in Shiga toxin transport. First, we wanted to confirm that GA activates p38 in HEp-2 cells. Indeed, GA treatment led to a strong and persistent increase in p38 phosphorylation that lasted at least 60 min after addition of the drug (Fig 6A). Thus, p38 is highly activated at the time of Shiga toxin addition in the transport assays.

The GA-induced p38 activation was counteracted by the p38 inhibitor SB203580 (Fig 6B), and we therefore performed the sulfation assay in the presence of both GA and SB203580. The combination of GA and SB203580 significantly reduced the GA-induced increase in Shiga toxin sulfation (Fig 6C), suggesting that p38 activity is at least partially responsible for the increased toxin transport. Similar results were obtained with the second generation inhibitor NVP-AUY922 (S1 Fig).

**Fig 7:**
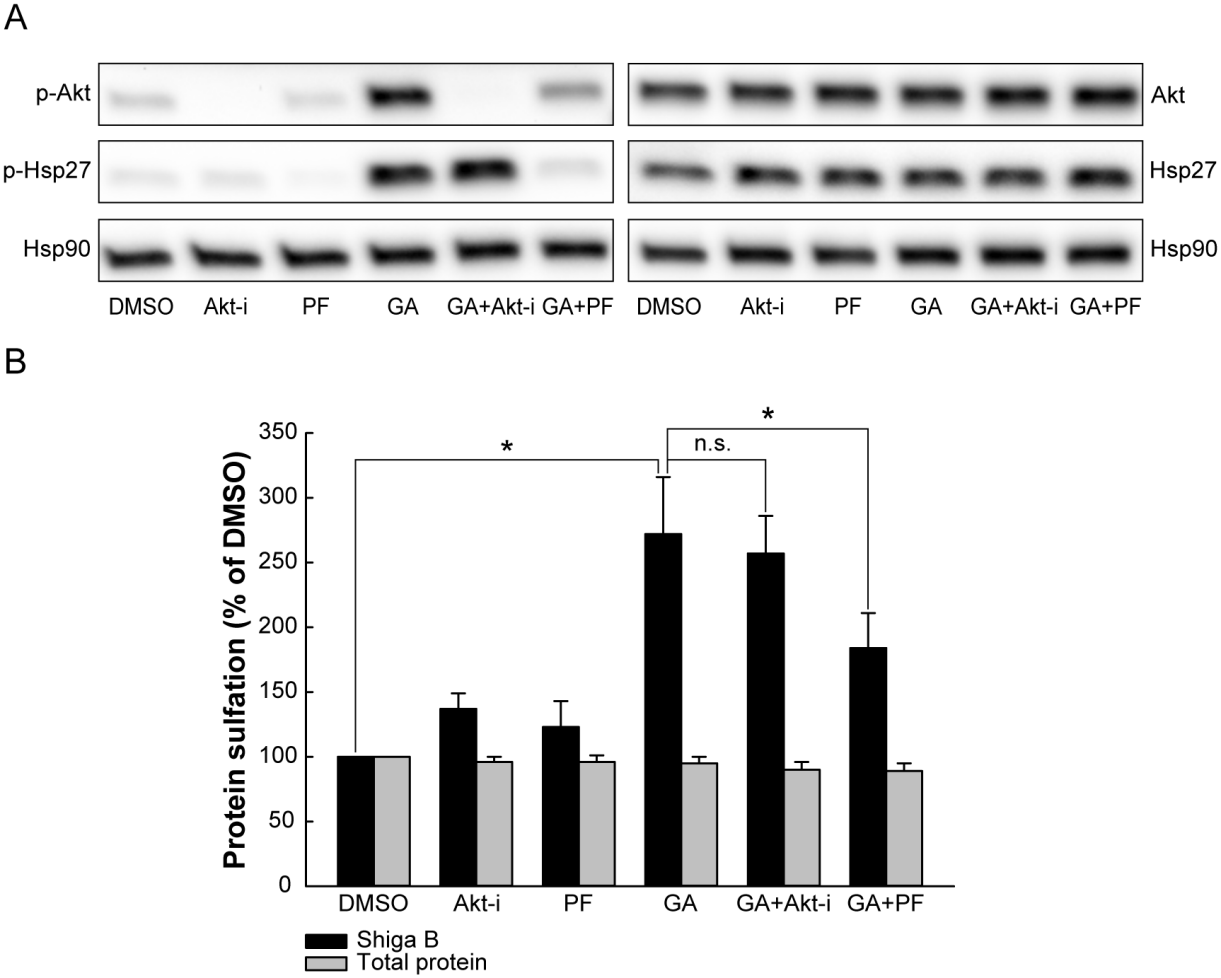
MK2 activation contributes to the increased retrograde transport after GA treatment. (A) HEp-2 cells were starved in sulfate-free medium for 3 h at 37°C, and during the last 30 min 2.5 μM Akt inhibitor VIII (Akt-i), 2.5 μM PF 3644022 (PF) or 10 μM GA were added. The cells were lysed and proteins were separated by SDS-PAGE. Blots were probed with the indicated antibodies, and Hsp90 was used as a loading control. (B) Cells were preincubated with 10 μM GA in combination with 2.5 μM Akt inhibitor VIII (Akt-i), or 2.5 μM PF 3644022 (PF) for 30 min and subsequently incubated with Shiga B-sulf2 for 1 h. The Shiga B sulfation (black bars) and total protein sulfation (grey bars) are expressed relative to control treatment (DMSO) and are plotted as mean values + SEM, *n* = 6. * p ≤ 0.05, paired Student’s *t*-test.

p38 activates a number of substrates, but the mitogen-activated protein kinase-activated protein kinase 2 (MAPKAPK-2 or MK2) is believed to be one of the most important kinases activated by p38 due to its role in mediating cellular stress and inflammatory responses [27] Under normal growth conditions, p38 constitutively forms a signaling complex with MK2, Akt and Hsp27. Upon activation, p38 phosphorylates MK2, which subsequently phosphorylates Hsp27, leading to the dissociation of Hsp27 from the complex [28–30]. The phosphorylation state of Hsp27 appears to be important for its role in the regulation of the actin cytoskeleton [31]. We found that both Hsp27 and Akt were phosphorylated after GA treatment (Fig 7A). To test whether the p38-MK2 pathway is important for retrograde transport, we performed the sulfation experiment in the presence of GA and the MK2 inhibitor PF 3644022 (Fig 7B). As Hsp27 has been reported to dissociate from the signaling complex also after Akt-mediated phosphorylation [28], we included the Akt inhibitor VIII in our experiments. While MK2 inhibition gave a strong reduction in the GA-induced phosphorylation of Hsp27, Akt inhibition did not prevent Hsp27 activation (Fig 7A). Interestingly, the MK2 inhibitor gave a similar reduction in the GA-induced increase in Shiga toxin transport as p38 inhibition, whereas Akt inhibition did not alter the GA-induced effect (Fig 7B). Thus, it seems that GA-induced p38 phosphorylation leading to activation of MK2 is involved in retrograde transport of Shiga toxin.

## Discussion

In this study we have investigated the effect of the Hsp90 inhibitor GA on retrograde transport to the Golgi apparatus. We show that GA treatment strongly enhances the transport of Shiga toxin to the Golgi apparatus, as measured by the sulfation assay and immunofluorescence. The increased Shiga toxin transport seems to be partially mediated by activation of the p38-MK2 pathway, as the GA-induced increase in Shiga toxin sulfation was partially counteracted by the p38 inhibitor SB203580 as well as the MK2 inhibitor PF3644022.

It is well known that GA promotes degradation of the ErbB2 receptor, presumably by altering endosomal sorting into the degradative pathway [6,7]. A recent study on HeLa cells showed that GA induced abnormally elongated endosomal structures with or without terminal multivesicular body (MVB) domains [7]. Interestingly, these morphological changes of endosomal compartments were suggested to also cause missorting of Hsp90-independent cargo, since GA treatment rerouted transferrin from early endosomes/recycling endosomes to MVBs and lysosomes [7]. This notion, that GA alters transport at the endosomal level, is also supported by our data obtained with HEp-2 cells. GA treatment strongly increased the Golgi transport of both Shiga toxin and ricin without increasing the endocytic uptake of the toxins, and the retrograde retrieval of the CI-M6PR was slightly increased, suggesting that GA has a more general effect on retrograde sorting processes. Importantly, the increased retrograde transport does not seem to be caused by a GA-induced effect on other pathways, such as reduced recycling of toxins to the plasma membrane or reduced degradation, which would lead to a net accumulation of toxin molecules in the early-recycling endosomes, and potentially, an increased transport into the retrograde pathway. However, it should be emphasized that the retrograde pathway is a specialized pathway requiring strict sorting for entry. This can be illustrated by the fact that ricin labeled with colloidal gold or multivalently coupled to HRP or nanoparticles is unable to enter the retrograde pathway, and the toxin is redirected to lysosomes [32,33]. Thus, since the retrograde pathway does not seem to be a default transport pathway, it is not given that a perturbation in another pathway would lead to increased retrograde transport.

In contrast to our data showing a GA-mediated increase in toxin transport to the Golgi, it was previously reported that Hsp90 inhibition in HeLa cells had no effect on the retrograde transport of ricin to the cytosol, but rather increased toxicity by preventing inactivation of the ricin A-chain present in the cytosol [34]. Moreover, GA was reported to block the retrotranslocation of cholera toxin to the cytosol without affecting its retrograde transport [35]. Although these data seem to be in disagreement with our findings, it should be emphasized that none of these studies specifically measured toxin transport to the Golgi apparatus and that they were performed in other cell lines. For ricin, the lag time before onset of toxicity was used as a measure of transport [34]. For cholera toxin, the amount of cholera toxin A1(CTA1) secreted into the extracellular medium was used to estimate intracellular transport [35]. For CTA1 secretion to occur, cholera toxin must first be transported to the ER, where CTA1 dissociates from the holotoxin [35]. Although toxin trafficking to the ER or cytosol was not significantly altered by GA treatment, this does not exclude an effect on retrograde sorting to the Golgi apparatus, as transport from the Golgi apparatus to the ER or retrotranslocation across the ER membrane may be rate-limiting transport steps.

Although GA has been suggested to alter endosomal sorting of Hsp90-dependent and-independent cargo due to ultrastructural changes of endosomal compartments, the mechanism behind the change of endosome morphology is still unclear [7]. Shiga toxin is transported retrogradely bound to its receptor Gb3. This receptor does not traverse the membrane and is not in contact with the cytosol. Thus, a direct interaction with Hsp90 is unlikely. We therefore looked for potential Hsp90 client proteins that could regulate retrograde transport. p38 has previously been shown to interact with the Hsp90-Cdc37 chaperone complex, and upon Hsp90 inhibition, p38 was released from the complex and activated by autophosphorylation [25]. In agreement with this, we found that GA rapidly activates p38 in HEp-2 cells and the activation persists well after the time-point when Shiga toxin is added. Earlier studies have shown that chemical inhibition or RNAi-mediated downregulation of p38 impairs retrograde transport of Shiga toxin [18], and in agreement with these data, inhibition of p38 after GA treatment gave a partial reduction in Shiga toxin sulfation, indicating a role for p38 in the increased retrograde transport after Hsp90 inhibition. This is also supported by the finding that the NVP-AUY922-mediated increase in Shiga toxin transport is completely negated by p38 inhibition.

**Fig 8:**
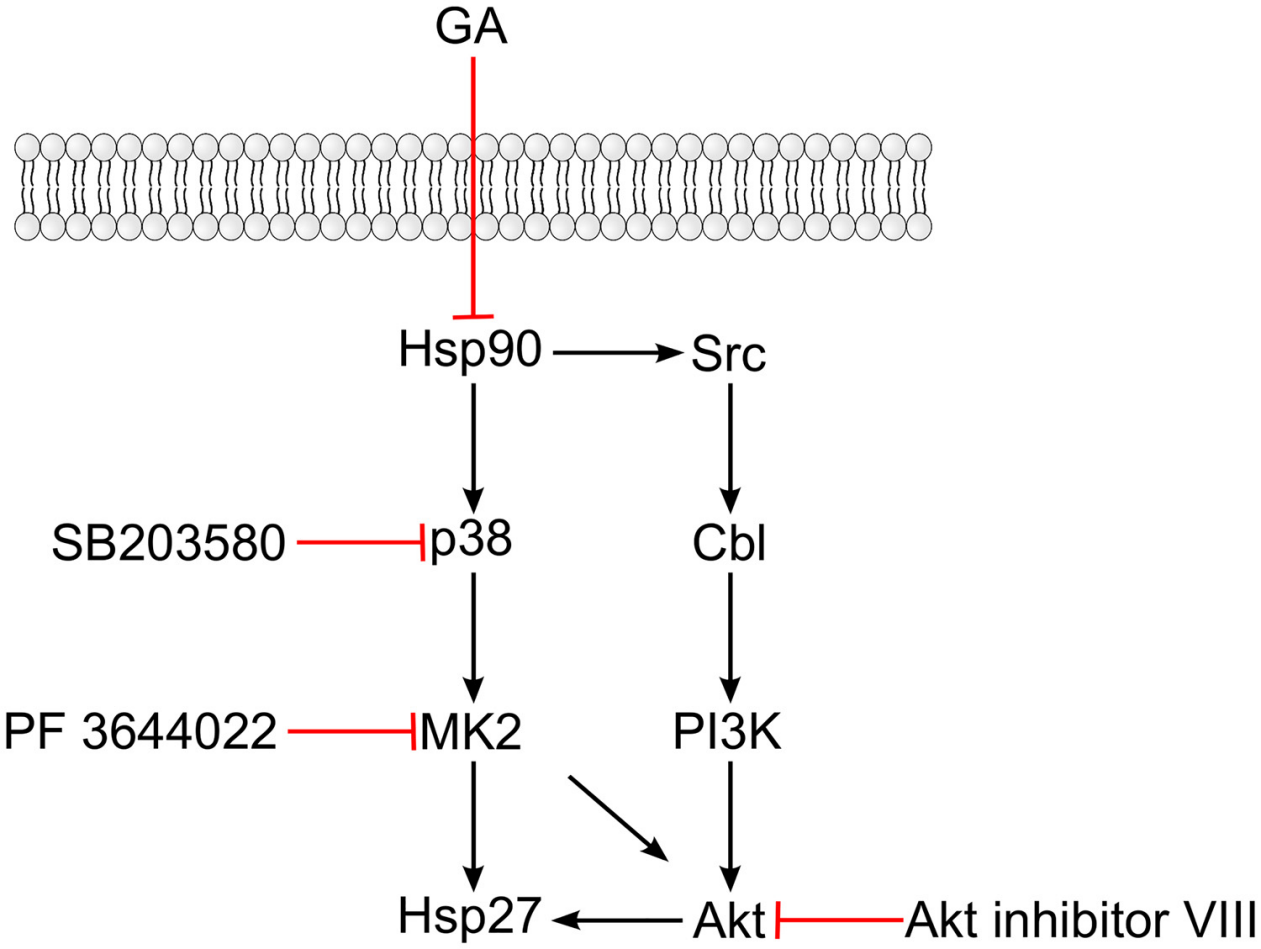
GA-induced activation of Hsp27 and Akt. Inhibition of Hsp90 by GA releases p38 from the Hsp90-Cdc37 complex, allowing it to autophosphorylate. Activated p38 phosphorylates MK2, which subsequently phosphorylates Hsp27. GA also leads to the dissociation of Src from Hsp90, resulting in transient activation. Src then phosphorylates Cbl, which recruits and activates phosphatidylinositol 3-kinase (PI-3K). Activation of PI-3K eventually leads to the activation of Akt. Akt can also be activated by MK2 and activated Akt has been shown to activate Hsp27. The illustration is based on references [25,28–30,37,39]

It is currently unclear how p38 activity might alter retrograde transport, and in an attempt to elucidate this, we investigated the role of the p38 substrate MK2. Together with p38, Akt and Hsp27, MK2 constitute a signaling complex important for controlling stress-induced apoptosis and actin remodeling [28]. Interestingly, inhibition of MK2 upon GA treatment gave a similar effect on Shiga toxin sulfation as p38 inhibition, suggesting that p38 mediates its effect via activation of MK2. In contrast, Akt inhibition upon GA treatment did not prevent Hsp27 phosphorylation, nor did it alter the GA-induced increase in retrograde transport. Akt is a client protein of Hsp90 which is degraded upon Hsp90 inhibition [36], but it is first transiently phosphorylated in a Src-dependent manner [37]. Thus, Akt activation by GA does not necessarily rely on p38, and based on our data using the Akt inhibitor, Akt activity does not seem to be crucial for Hsp27 phosphorylation after GA treatment. The GA-mediated activation of Hsp27 and Akt is summarized in Fig 8. In the signaling complex, Hsp27 is normally present as an oligomer [28]. Upon activation, phosphorylated monomeric Hsp27 is released from the complex, which is associated with actin reorganization. In unstressed cells, non-phosphorylated Hsp27 monomers bind to the plus-end of F-actin and act as actin-capping proteins, preventing actin polymerization. Non-phosphorylated oligomers and phosphorylated monomers of Hsp27 have reduced affinity for the plus-end of F-actin, and phosphorylated Hsp27 monomers are instead thought to bind to the sides of F-actin, thus stabilizing the microfilaments [31,38]. Interesting in this context, is the previous finding that the actin-stabilizing drug Jasplakinolide increases transport of Shiga toxin to the Golgi apparatus [15].

The binding of phosphorylated Hsp27 to the sides of F-actin was suggested to be a long-term effect of Hsp27 activation after heat shock, however, a 1h treatment with arsenite was shown to be sufficient to protect cells against the actin depolymerizing drug cytochalasin D in an Hsp27-dependent manner [31,40]. It is not known whether a short-term incubation with GA would lead to Hsp27-mediated actin stabilization, and to further complicate the picture, Hsp27 is not the only actin-regulating protein that is affected by GA. Treatment with GA or its derivative 17-AAG was shown to stimulate actin stress fiber formation in a Rho- and ROCK dependent manner, and although Rho was activated after 30 min of GA treatment, the effect on actin stress fibers was not visible until after 2 h of treatment [41].

It should be noted that p38 activation also regulates the membrane association of Rab5, one of the key regulators of early endocytic traffic, and its effectors EEA1 and rabenosyn-5 [42,43]. This may be relevant for retrograde transport as depletion of rabenosyn-5 was found to disrupt Shiga toxin transport to the Golgi [44]. However, since inhibition of MK2 or p38 counteracts the GA-induced increase in Shiga toxin transport to the same extent, it is more likely that the contribution of p38 activation is mediated via the p38-MK2 pathway.

Although p38 contributes to the increased retrograde transport of Shiga toxin after GA treatment, inhibition of p38 does not completely counteract the GA-mediated effect, suggesting that Hsp90 inhibition alters more than one process important for retrograde transport. This is supported by the finding that p38 inhibition fails to reduce the increased Golgi transport of ricin after GA treatment (S4 Fig). However, it should be noted that in contrast to retrograde transport of Shiga toxin, ricin transport does not seem to be p38 dependent [18]. Moreover, radicicol did not induce p38 phosphorylation (S5 Fig), further supporting the existence of additional mechanisms. Although geldanamycin and radicicol would be expected to have similar effects, combination of the two drugs were shown to have a synergistic inhibitory effect on glucocorticoid receptor-dependent transcription and hormone binding [45]. The synergy was not related to the ATPase activity of Hsp90, but was suggested to be caused by subtle differences in drug binding to the ATPase binding pocket of Hsp90 [46].

Hsp90 has been suggested to have different cellular functions under normal growth conditions and during environmental stress. In a genome-wide chemical-genetic screen in yeast, deletion strains of components in the gene ontology (GO) categories vesicle-mediated transport and the Golgi apparatus were hypersensitive to Hsp90 inhibition under normal growth conditions, whereas components of the cell cycle, meiosis and cytokinesis were more profoundly affected at elevated temperatures [47]. Under normal growth conditions, several multi-subunit complexes, including the conserved oligomeric Golgi (COG) complex, the endosomal sorting complex required for transport (ESCRT) I, II, and III, and the retromer complex, were among the most prominent Hsp90 targets. Interestingly, RNAi-mediated knockdown of COG complex- and retromer subunits has previously been shown to impair retrograde transport of Shiga toxin [48–52]. To our knowledge, it has not been studied whether short-term Hsp90 inhibition by GA would affect the COG and retromer complexes and whether this may be important for retrograde transport. However, short-term Hsp90 inhibition did not seem to change the distribution of the retromer component sorting nexin 1 (SNX1) (S6 Fig).

In conclusion, we have demonstrated that the Hsp90 inhibitor GA enhances retrograde transport of Shiga toxin and ricin, and also to some extent retrograde retrieval of CI-M6PR. GA-induced activation of p38 and MK2 seems to contribute to the increased transport of Shiga toxin, but additional mechanisms are likely to exist.

## Materials and Methods

### Reagents and antibodies

H_2_^35^SO_4_ was from Hartman Analytics. The plasmids expressing the non-toxic Shiga toxin 1 mutant and Shiga B-sulf2 were kind gifts from Dr. A.D. O’Brien (Uniformed Services University of the Health Sciences, Bethesda, Maryland, USA), and Dr. B. Goud (Institut Curie, Paris, France), respectively. Shiga toxin 1 mutant was produced and purified as described in [21] and Shiga B-sulf2 was prepared as described below. A modified ricin A chain containing a tyrosine sulfation site was produced, purified and reconstituted with ricin B to form ricin-sulf1 as previously described [53]. Ricin holotoxin was from Sigma-Aldrich. The cell line stably expressing the CD8-M6PR fusion protein was a kind gift from Dr. M.N.J. Seaman (Cambridge Institute for Medical Research, Cambridge, UK). Geldanamycin, radicicol and SB203580 were from Sigma-Aldrich. The mesylate salt of NVP-AUY922 was provided by Novartis. Akt inhibitor VIII was from Calbiochem and PF 3644022 was from Tocris Bioscience. The following antibodies were used: Monoclonal mouse anti-Shiga toxin STX1-3C10 and STX1-13C4 (Toxin Technology), polyclonal rabbit anti-lectin (*Ricinus Communis*, R1254, Sigma-Aldrich), polyclonal rabbit anti-giantin (PRB-114C, BioLegend/Covance Antibody Products), monoclonal mouse anti-CD8 (Sigma-Aldrich), monoclonal mouse anti-Hsp90 (610419), monoclonal mouse anti-SNX1 (611482), monoclonal mouse anti-p38 (612168, BD Transduction Laboratories), polyclonal rabbit anti-EEA1 (2411S), polyclonal rabbit anti-phospho-p38 (9211), monoclonal rabbit anti-phospho-Akt (4058), polyclonal rabbit anti-Akt (9272), polyclonal rabbit anti-phospho-Hsp27 (2401), monoclonal mouse anti-Hsp27 (2402, Cell Signaling), polyclonal Alexa-568 secondary antibody (A10037, Molecular probes), and polyclonal HRP-, Alexa-488- and Cy3-conjugated secondary antibodies from Jackson Immunoresearch (115-035-003, 111-035-144, 711-545-152, 115-165-146, 715-165-151). Other chemicals used were from Sigma-Aldrich unless otherwise stated.

### Cell lines

HEp-2 (human epidermoid laryngeal carcinoma) cells (ATCC: CCL-23) and HeLa (human cervical adenocarcinoma) cells stably expressing the CD8-M6PR fusion protein (from Dr. M.N.J. Seaman, [23]) were grown at 5% CO_2_ in Dulbecco’s Modified Eagle Medium (DMEM; Invitrogen) with 10% v/v fetal calf serum (FCS; PAA Laboratories) supplemented with 100 U/ml penicillin and 100 U/ml streptomycin (Invitrogen). Cells were seeded one day prior to experiments.

### Preparation of Shiga B-sulf2

A modified version of the Shiga toxin B-subunit containing C-terminal tandem sulfation sites (Shiga B-sulf2) was produced in *Escherichia coli* BL21 (DE3) cells. LB medium supplemented with ampicillin (100 μg/ml) was inoculated 1:100 with an overnight culture and incubated for approximately 16 h at 30°C. Protein expression was induced by changing the culture temperature to 42°C for 3h. Cells were harvested by centrifugation. The cell pellet was resuspended in 25% sucrose, 1 mM Na_2_EDTA, and 20 mM Tris-HCl, pH 8.0; and gently shaken at 25°C for 20 min. Cells were spun down, resuspended in ice-cold distilled water and incubated on ice for 10 min. After centrifugation, ammonium sulfate was added to the supernatant at a concentration of 60% saturation and the solution was incubated at 25°C for 1 h. Proteins were sedimented by centrifugation at 13,000 g for 15 min. The protein pellet was resuspended in a buffer containing 1 M ammonium sulfate and 50 mM sodium phosphate, pH 8. The sample was centrifuged at 13,000 g for 10 min and the supernatant was filtered through a 0.45 μM filter. Proteins were separated on a HiTrap Butyl HP column (GE Healthcare) using 50 mM sodium phosphate (pH 8.0) as mobile phase and a segmented gradient of ammonium sulfate from 1–0.45 M in 2 column volumes (CV), 0.45 M for 5 CV, 0.45–0.40 M in 2 CV, 0.40 M for 2 CV, and 40–0 M in 2 CV. Fractions containing Shiga B-sulf2 were pooled, the buffer was changed by ultrafiltration using Amicon Ultra filters with molecular weight cut-off of 3 kDa (Millipore) and the retained sample was loaded onto a MonoQ 5/50 GL column (GE Healthcare). The proteins were separated using a mobile phase of 20 mM Tris, pH 8.0 and a segmented gradient of NaCl: 0–0.58 M in 5 CV, 0.58–0.61 M in 10 CV, and 0.61–1M in 2 CV. Fractions containing Shiga B-sulf2 were pooled and the concentration of NaCl was reduced by buffer change using Amicon Ultra filters with molecular weight cut-off of 3 KDa (Millipore). The resulting sample was purified a second time over the MonoQ 5/50 GL column using the same settings as described above. The fractions containing Shiga B-sulf2 were pooled, the buffer was changed to 20 mM Tris, pH 8.0 and the sample was concentrated by ultrafiltration. The purity of the Shiga B-sulf2 preparation was assessed by Coomassie staining following SDS-PAGE on a 4–20% polyacrylamide gel. The result showed only the band corresponding to the Shiga B-sulf2 subunits of the Shiga B-sulf2 complex (S7 Fig).

### Endocytosis of Shiga toxin and ricin

The endocytosis of Shiga toxin was quantified as previously described [15]. Briefly, after inhibitor treatment cells were incubated with 40 ng/ml of Shiga toxin 1 mutant labeled with biotin bound via a reducible linker (EZ-link Sulfo-NHS-SS-Biotin, Pierce Biotechnology) for 20 min at 37°C. The cells were then washed with cold buffer (0.14 M NaCl, 2 mM CaCl_2_, 20 mM HEPES, pH 8.6). To determine the amount of internalized toxin, half of the plate was incubated with 0.1 M sodium 2-mercaptoethanesulfonate (MESNa) and 2 mg/ml BSA in the same buffer on ice to reduce the SS-biotin in cell surface-bound toxin. The other half of the plate was mock treated to determine the amount of total cell-associated toxin (internalized + cell surface-bound toxin). The cells were washed and lysed in a lysis buffer (0.1 M NaCl, 10 mM Na_2_HPO_4_ (pH 7.4), 1 mM EDTA, 1% Triton X-100, supplemented with a mixture of Complete protease inhibitors (Roche Diagnostics) and 60 mM *n*-octyl-β-pyranoside). Cell lysates were incubated in the presence of 0.5 μg/ml BV-TAG-labeled monoclonal anti-Shiga toxin antibody (3C10) containing a Tris(bipyridine)-chelated ruthenium (II) atom (BioVeris Corporation) and 0.1 mg/ml streptavidin-coated Dynabeads (Invitrogen) for 1.5 h in assay diluent (0.2% BSA, 0.5% Tween20 in PBS) with gentle shaking. The amount of streptavidin-captured BV-TAG-labeled anti-Shiga toxin was determined by the specialized electro-chemiluminescent detection system M1R Analyzer (BioVeris Corporation).

Ricin was ^125^I-labeled using the IODO-GEN Iodination Reagent (Pierce Biotechnology) according to the manufacturer’s protocol. Cells were treated with or without 10 μM GA for 30 min at 37°C and subsequently incubated with ~50 ng/ml ^125^I-labeled ricin for 30 min. To distinguish between total cell-associated toxin and internalized toxin, half of the samples were incubated with 0.1 M lactose for 5 min at 37°C to remove cell surface-bound ricin. The cells were then washed in PBS or 0.1 M lactose, dissolved in 0.1 M KOH, and the amount of total cell-associated or internalized toxin was measured using an LKB Wallac 1261 Multigamma γ-counter (LKB Instruments).

### Sulfation of Shiga B-sulf2 or ricin-sulf1

The cells were washed twice with sulfate-free medium and subsequently incubated with 0.2 mCi/ml ^35^SO_4_^2-^ for 3 h at 37°C in the same medium, with or without inhibitors as indicated in the figure legends. Then ~2 μg/ml Shiga B-sulf2 or ~4 μg/ml ricin-sulf1 was added and the incubation continued for 1 h or 1.5 h. Cells treated with ricin-sulf1 were subsequently incubated twice with 0.1 M lactose in HEPES-buffered medium for 5 min at 37°C to remove surface-bound toxin. The cells were then washed with ice-cold PBS and lysed (0.1 M NaCl, 10 mM Na_2_HPO_4_ (pH 7.4), 1 mM EDTA, 1% Triton X-100, supplemented with a mixture of Complete protease inhibitors (Roche Diagnostics) and 60 mM *n*-octyl-β-pyranoside). Shiga B-sulf2 or ricin-sulf1 was immunoprecipitated from cleared lysates overnight at 4°C using Protein A Sepharose beads (GE Healthcare) with the appropriate antibody adsorbed. The immunoprecipitate was washed twice with 0.35% Triton X-100 in PBS, resuspended in sample buffer and boiled. The immunoprecipitate was separated by SDS-PAGE, transferred to a PVDF membrane and investigated by digital autoradiography using a phosphor imaging screen (Imaging Screen-K (Kodak), Bio-Rad Laboratories Inc). Images were acquired using the Molecular Imaging PharosFX System (Bio-Rad Laboratories Inc) and band intensities were quantified with the Quantity One 1-D Analysis Software (Bio-Rad Laboratories Inc). The total amount of sulfated proteins was determined by TCA precipitation of the remaining lysates followed by β-counting.

### Shiga toxin recycling

To measure Shiga toxin recycling, a modified version of the endocytosis method described above was used. The cells were incubated with or without 10 μM GA in HEPES-buffered medium for 30 min at 37°C before addition of 40 ng/ml of biotinylated Shiga toxin 1 mutant labeled with the reducible SS-Biotin and the incubation was continued for 30 min at 37°C. The cells were then washed with cold buffer (0.14 M NaCl, 2 mM CaCl_2_, 20 mM HEPES, pH 8.6) and treated with 0.1 M MESNa and 2 mg/ml BSA in the same buffer for 30 min at 4°C. This is to remove the SS-biotin from Shiga toxin present at the cell surface to prevent its detection. The cells were subsequently washed with warm HEPES-buffered medium and chased in the same medium with or without GA for 30 min at 37°C. Then, the medium was collected and detached cells were removed by centrifugation. The cells were washed with cold buffer and half of the 24 well plate was treated with 0.1 M MESNa and 2 mg/ml BSA in the same buffer to remove SS-biotin from recycled Shiga toxin present at the cell surface. The other half of the plate was mock treated to determine the amount of total cell-associated Shiga toxin. The cells were lysed in lysis buffer (0.1 M NaCl, 10 mM Na_2_HPO_4_ (pH 7.4), 1 mM EDTA, 1% Triton X-100, supplemented with a mixture of Complete protease inhibitors (Roche Diagnostics) and 60 mM *n*-octyl-β-pyranoside) and cell lysates and medium were incubated in the presence of BV-TAG-labeled monoclonal anti-Shiga toxin antibody containing a Tris(bipyridine)-chelated ruthenium (II) atom and 0.1 mg/ml streptavidin-coated Dynabeads and analyzed as described above in the endocytosis method. The amount of recycled Shiga toxin was determined as the amount of Shiga toxin in medium + the difference between internalized and total cell-associated Shiga toxin divided by the total amount of Shiga toxin (medium + total cell-associated Shiga toxin).

### Ricin degradation and recycling

Cells were incubated with or without 10 μM GA for 30 min at 37°C before ^125^I-ricin (100–500 ng/ml) was added and the incubation continued for 20 min at 37°C. The cells were then incubated with 0.1 M lactose in HEPES-buffered medium for 5 min and washed three times in the same solution to remove surface-bound ricin. The cells were subsequently chased in 1 mM lactose in HEPES-buffered medium with or without 10 μM GA for 2 h at 37°C. 1 mM lactose was included to prevent re-binding and reuptake of recycled ricin. The medium was collected and proteins were precipitated with 0.5 mg/ml BSA and 5% TCA and pelleted by centrifugation. The cells were dissolved in 0.1 M KOH. The radioactivity associated with the medium supernatant and pellet, and with the cells was measured with a LKB Wallac 1261 Multigamma γ-counter. Degradation or recycling was calculated as the amount of radioactivity in the non-precipitable fraction of the medium (supernatant) or the precipitable fraction of the medium (pellet), divided by the total radioactivity in cells and in medium, respectively.

### CI-M6PR retrograde transport

CI-M6PR transport was studied in a HeLa cell line stably expressing the CD8-M6PR fusion protein. The protocol was slightly modified from Breusegem and Seaman [22]. Cells grown on glass coverslips were incubated with 10 μM GA for 30 min in HEPES-buffered medium at 37°C. Then, the cells were chilled in cold HEPES-buffered medium for 10 min at 4°C to stop trafficking. The cells were labeled with 10 μg/ml CD8 antibody for 30 min at 4°C before washing in cold PBS and chasing in warm HEPES-buffered medium in the presence of inhibitor for 0 or 15 min. The cells were washed and prepared for immunofluorescence confocal microscopy as described below. The following antibodies were used: 1:1000 anti-giantin, 1:200 Alexa488 donkey anti-rabbit IgG and 1:500 Cy3 donkey anti-mouse IgG or Alexa568 donkey anti-mouse IgG.

### Immunofluorescence confocal microscopy

Cells grown on glass coverslips were serum-starved in HEPES-buffered medium for 2 h before incubation with 10 μM GA for 30 min. For retromer localization studies, the cells were subsequently fixed, while in Shiga toxin experiments, 100 ng/ml Shiga toxin 1 mutant was added and the incubation continued for 30 min. All samples were fixed in 10% formalin (Sigma-Aldrich) or 3% methanol-free paraformaldehyde (Alfa Aesar) and permeabilized with 0.1% Triton X-100, before blocking in 5% FCS. The samples were incubated with primary antibodies in 5% FCS for 1 h at room temperature (for Shiga toxin experiments: 4 μg/ml STX1-3C10 and 1:1000 anti-giantin, for retromer localization studies: 1:200 anti-SNX1 and 1:100 anti-EEA1), followed by 30 min incubation with fluorophore-conjugated secondary antibodies (1:200 Alexa488 donkey anti-rabbit IgG and 1:500 Cy3 goat or donkey anti-mouse IgG for Shiga toxin experiments and retromer localization studies, respectively). The samples were mounted in ProLong Gold with DAPI (Molecular Probes) and investigated using a Zeiss LSM 780 laser scanning confocal microscope (Carl Zeiss MicroImaging) equipped with an Ar-Laser Multiline (458/488/514 nm), a DPSS-561 10 (561 nm), and a Laser diode 405–30 CW (405 nm). The objective used was a Zeiss Plan-Apochromat 63×/1.40 Oil DIC M27. Images were acquired using the ZEN 2010 software (Carl Zeiss MicroImaging). Fiji software [54] was used for quantification of signal intensities and for image preparation. Shiga toxin and CD8-M6PR transport to the Golgi was determined in a similar manner as described in Breusegem and Seaman [22]. A Golgi mask was created from the giantin staining, and the cell outline was defined from composite images with increased contrast. The intensity of Shiga toxin or CD8-M6PR in the cell outline and in the Golgi mask was then measured from background-subtracted images and transport was calculated as the ratio of signal intensity in the Golgi mask to the signal in the whole cell mask. As the ratio of CD8-M6PR reaching the Golgi varied in different experiments, data was normalized to DMSO samples in individual experiments. For the retromer localization studies, the colocalization between SNX1 and EEA1 was determined by using the coloc2 plugin in Fiji to measure Manders’ colocalization coefficients in single cells in background-subtracted images. For visualization, the image contrast was enhanced and the histogram normalized to contain 0.5% saturated pixels.

### Western blotting

Cells were serum-starved for 2–2.5 h and subsequently treated with inhibitors as indicated in the figure legends. The cells were washed and lysed in lysis buffer (0.1 M NaCl, 10 mM Na_2_HPO_4_ (pH 7.4), 1 mM EDTA, 1% Triton X-100, supplemented with a mixture of Complete protease inhibitors and PhosStop phosphatase inhibitors (Roche Diagnostics) and 60 mM *n*-octyl-β-pyranoside), before separation by SDS-PAGE and transfer to PVDF membranes. The membranes were blocked in 5% milk in TBS for 1 h, washed and incubated overnight with primary antibodies diluted in 5% BSA in 0.1% TBS-Tween (p-p38 1:1000, p38 1:1000, p-Akt 1:2000, Akt 1:1000, p-Hsp27 1:1000, Hsp27 1:1000, Hsp90 1:5000). The membranes were washed and incubated with appropriate HRP-labeled secondary antibodies (1:5000) in 5% milk in TBS-Tween for 1 h at room temperature. After washing, the membranes were incubated with SuperSignal West Dura Extended Duration Substrate (Thermo Scientific) for 5 min and images were acquired using the ChemiDoc XRS+ System with Image Lab Software (Bio-Rad Laboratories Inc).

### Statistics

All experiments were performed with duplicates. The experimental results are presented as mean values + standard error of the mean (SEM) or standard deviation (SD) of *n* independent experiments, where *n* is indicated in each figure legend. The paired Student’s *t*-test was used to determine the difference between means of two groups and the minimum level of significance was set at p ≤ 0.05.

## Supporting Information

S1 Fig Retrograde transport of Shiga toxin is increased upon Hsp90 inhibition by NVP-AUY922 and is negated by p38 inhibition. HEp-2 cells were treated with DMSO or 100 nM NVP-AUY922 alone or in combination with 10 μM SB 203580 (SB) for 30 min before 2 μg/ml Shiga B-sulf2 was added and the incubation continued for 1 h. The cells were lysed, and the toxin was immunoprecipitated and separated by SDS-PAGE. The amount of sulfated toxin and the total protein sulfation was determined as described in Materials and Methods. The toxin sulfation (black bars) and total protein sulfation (grey bars) are expressed relative to control treatment (DMSO) and are plotted as mean values + SD, *n* ≥ 2. * p ≤ 0.05, paired Student’s *t*-test.
(TIF)

S2 Fig GA does not alter ricin endocytosis. HEp-2 cells were preincubated with 10 μM GA for 30 min at 37°C and subsequently incubated with ~50 ng/ml ^125^I-labeled ricin for 30 min. The amount of internalized or total cell-associated toxin was quantified as described in Materials and Methods. Mean values + SEM of total cell-associated (black bars) and internalized (grey bars) ^125^I-labeled ricin are presented as percentage of control (DMSO), *n* = 2.
(TIF)

S3 Fig Ricin recycling and degradation is not altered by GA treatment. HEp-2 cells were preincubated with 10 μM GA for 30 min at 37°C and subsequently incubated with 100–500 ng/ml ^125^I-ricin for 20 min. Cell surface-associated ricin was removed by lactose washes and the toxin chased in the cells for another 2 h in the presence of inhibitor. The amount of cell-associated and released (precipitable and non-precipitable) ^125^I was measured to determine ricin recycling and degradation. (A) Ricin recycling was calculated as the precipitable fraction of ^125^I in the medium divided by the total amount of ^125^I. Mean values + SEM are presented as percentage of control (DMSO). (B) Ricin degradation was calculated as the non-precipitable fraction of ^125^I in the medium divided by the total amount of ^125^I. Mean values + SEM are presented as percentage of control (DMSO), *n* = 3.
(TIF)

S4 Fig The GA-mediated increase in ricin sulfation is not reduced after p38 inhibition. HEp-2 cells were preincubated with 10 μM GA in combination with 10 μM SB 203580 (SB) for 30 min and subsequently incubated with ricinsulf-1 for 1.5 h. The ricin sulfation (black bars) and total protein sulfation (grey bars) are expressed relative to control treatment (DMSO) and are plotted as mean values + SEM, *n* = 3. *** p ≤ 0.005, paired Student’s *t*-test.
(TIF)

S5 Fig Radicicol does not activate p38. HEp-2 cells were serum-starved in HEPES-buffered medium before incubation with 10 μM GA or 1 μM radicicol (Rad) for 30 min. The cells were lysed and proteins were separated by SDS-PAGE. The membranes were cut above the molecular marker for 150 kDa and just below 75 kDa, 50 kDa and 25 kDa. Blots were probed with the indicated antibodies. Hsp90 was used as a loading control.
(TIF)

S6 Fig SNX1 localization is not altered by GA treatment. (A) HEp-2 cells were treated with 10 μM GA for 30 min and subsequently fixed, permeabilized and stained with antibodies against SNX1 (magenta) and EEA1 (green). DAPI is shown in blue. Scale bar 20 μm. (B) The colocalization between SNX1 and EEA1 was quantified using the coloc2 plugin in the Fiji software and is presented as the mean Manders’ colocalization coefficient for the ratio of SNX1 colocalizing with EEA1 + SEM. *n* = 3, with at least 59 cells quantified for each condition. * p ≤0.05, paired Student’s *t*-test.
(TIF)

S7 Fig Purity of Shiga B-sulf2. Coomassie-stained SDS-polyacrylamide gel (4–20%) showing a single band for Shiga B-sulf2. Purified Shiga toxin 1 mutant was included as a reference to show the A-moiety (~32 kDa) and B-subunits (~8 kDa) of Shiga toxin. Shiga B-sulf2 runs slightly higher than the B-subunit due to the additional sulfation sites.
(TIF)

S8 Fig Original Western Blots. Original western blots for (A) Fig 6A, (B) Fig 6B, and (C) Fig 7A. The blots in A and B were cut between the molecular markers 150 and 250 kDa, 50 and 75 kDa, and 20 and 25 kDa. The blots in C were cut above 150 kDa and just below 75 kDa, 37 kDa and 15 kDa. The exposure time was optimized for each blot to give strong signals without saturation. The red boxes indicate the bands shown in Figs 6 and 7.
(TIF)
