# Supplementary figures and images for: Geldanamycin Enhances Retrograde Transport of Shiga Toxin in HEp-2 Cells
Source: PLoS One. 2015 May 27;10(5):e0129214. doi: 10.1371/journal.pone.0129214 (PMC4445914; doi:10.1371/journal.pone.0129214)

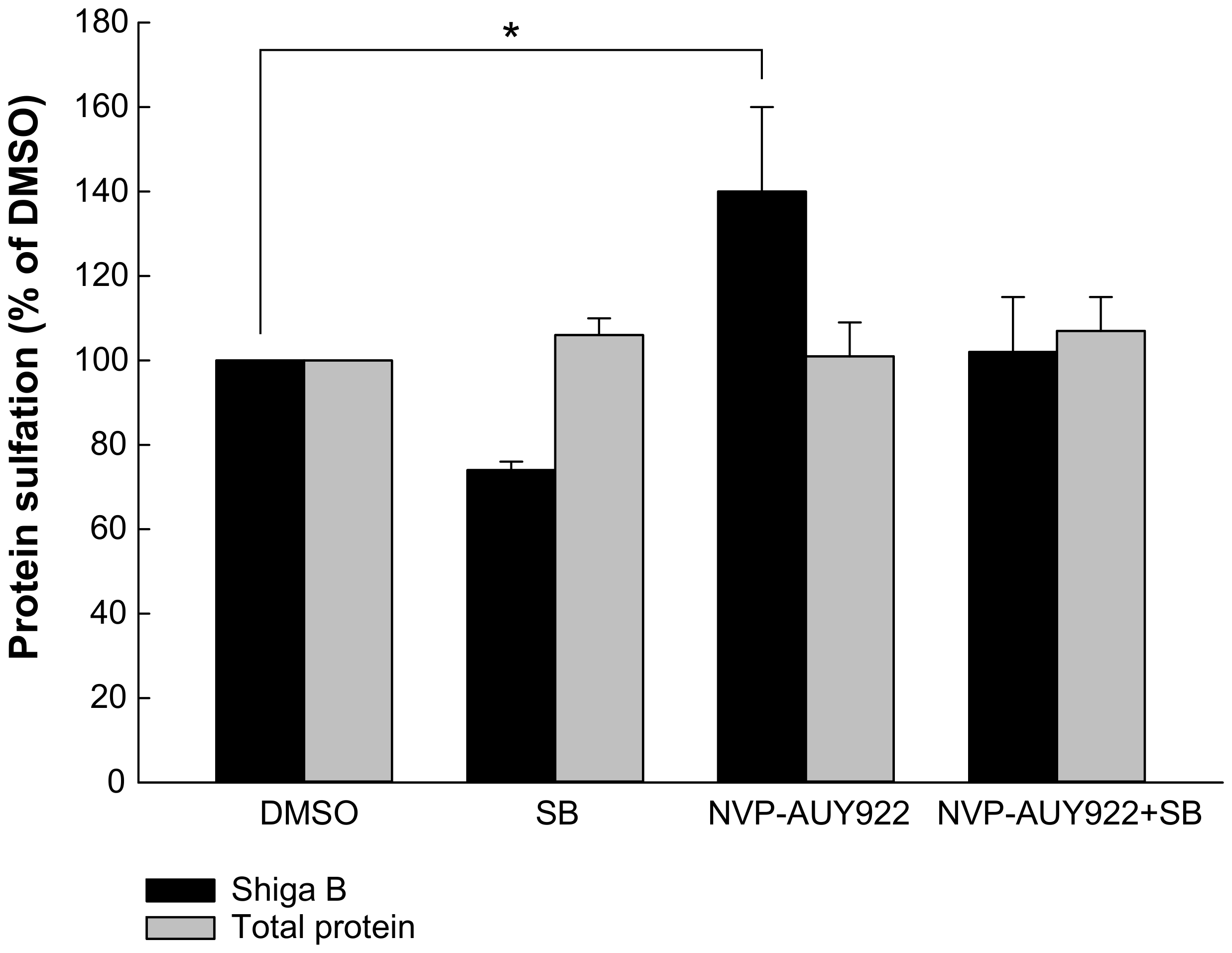

Supplement: S1 Fig — HEp-2 cells were treated with DMSO or 100 nM NVP-AUY922 alone or in combination with 10 μM SB 203580 (SB) for 30 min before 2 μg/ml Shiga B-sulf2 was added and the incubation continued for 1 h. The cells were lysed, and the toxin was immunoprecipitated and separated by SDS-PAGE. The amount of sulfated toxin and the total protein sulfation was determined as described in Materials and Methods. The toxin sulfation (black bars) and total protein sulfation (grey bars) are expressed relative to control treatment (DMSO) and are plotted as mean values + SD, n ≥ 2. * p ≤ 0.05, paired Student’s t-test. (TIF) [file pone.0129214.s001.tif]

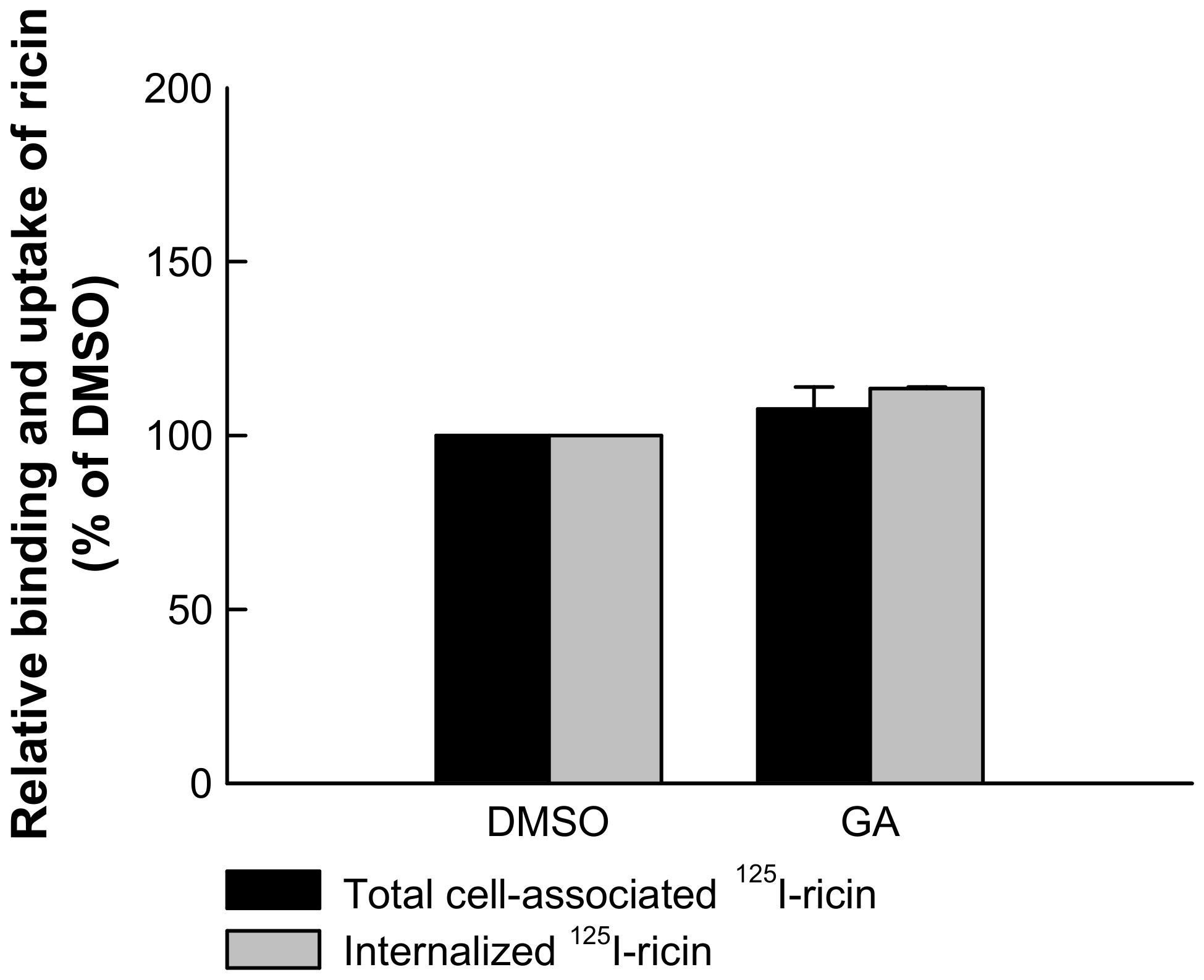

Supplement: S2 Fig — HEp-2 cells were preincubated with 10 μM GA for 30 min at 37°C and subsequently incubated with ~50 ng/ml 125I-labeled ricin for 30 min. The amount of internalized or total cell-associated toxin was quantified as described in Materials and Methods. Mean values + SEM of total cell-associated (black bars) and internalized (grey bars) 125I-labeled ricin are presented as percentage of control (DMSO), n = 2. (TIF) [file pone.0129214.s002.tif]

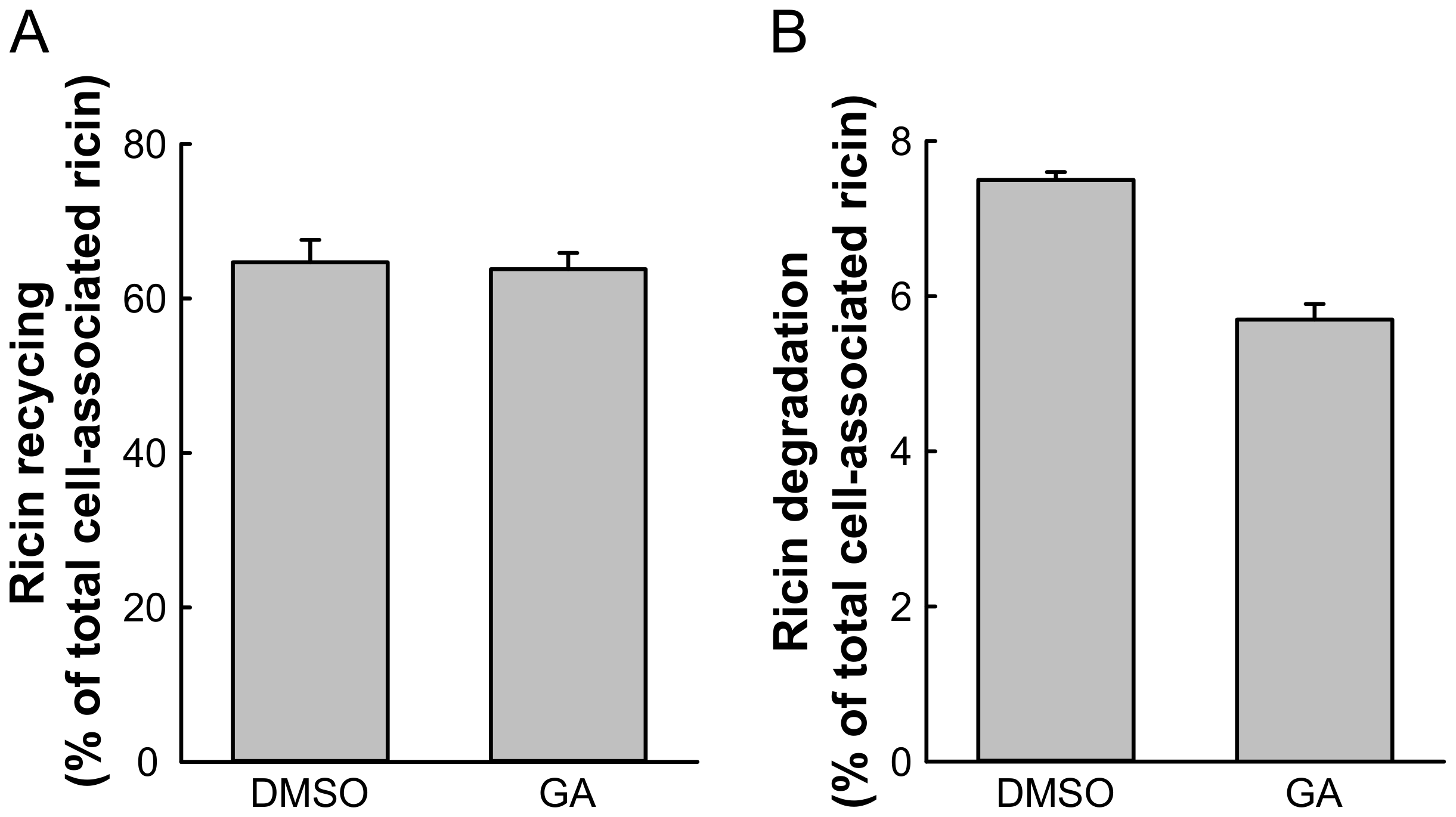

Supplement: S3 Fig — HEp-2 cells were preincubated with 10 μM GA for 30 min at 37°C and subsequently incubated with 100–500 ng/ml 125I-ricin for 20 min. Cell surface-associated ricin was removed by lactose washes and the toxin chased in the cells for another 2 h in the presence of inhibitor. The amount of cell-associated and released (precipitable and non-precipitable) 125I was measured to determine ricin recycling and degradation. (A) Ricin recycling was calculated as the precipitable fraction of 125I in the medium divided by the total amount of 125I. Mean values + SEM are presented as percentage of control (DMSO). (B) Ricin degradation was calculated as the non-precipitable fraction of 125I in the medium divided by the total amount of 125I. Mean values + SEM are presented as percentage of control (DMSO), n = 3. (TIF) [file pone.0129214.s003.tif]

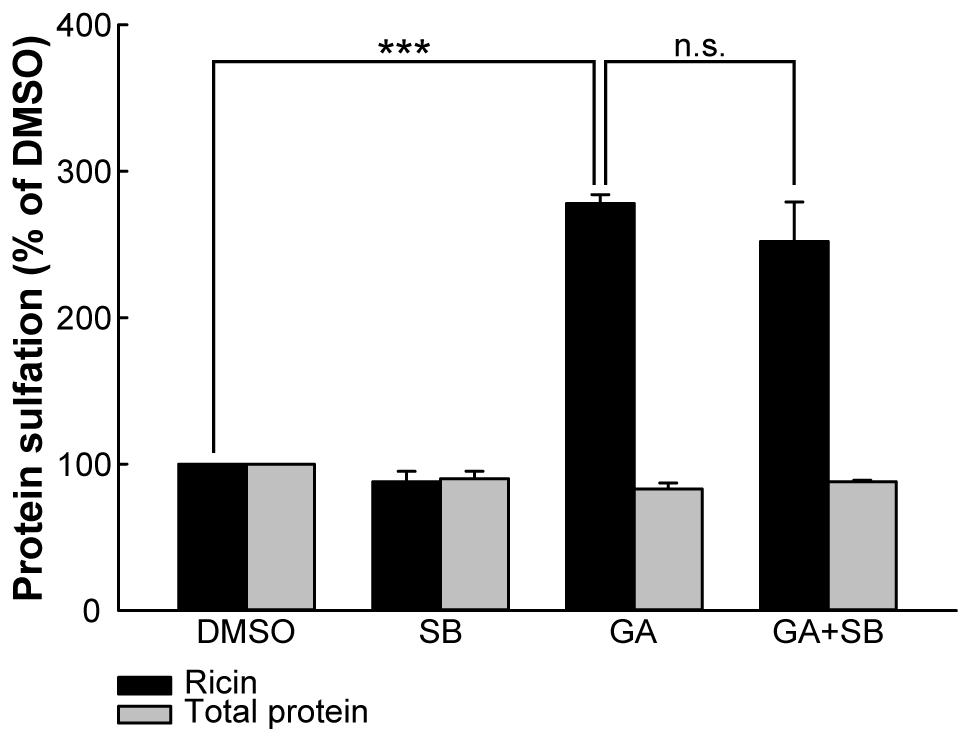

Supplement: S4 Fig — HEp-2 cells were preincubated with 10 μM GA in combination with 10 μM SB 203580 (SB) for 30 min and subsequently incubated with ricinsulf-1 for 1.5 h. The ricin sulfation (black bars) and total protein sulfation (grey bars) are expressed relative to control treatment (DMSO) and are plotted as mean values + SEM, n = 3. *** p ≤ 0.005, paired Student’s t-test. (TIF) [file pone.0129214.s004.tif]

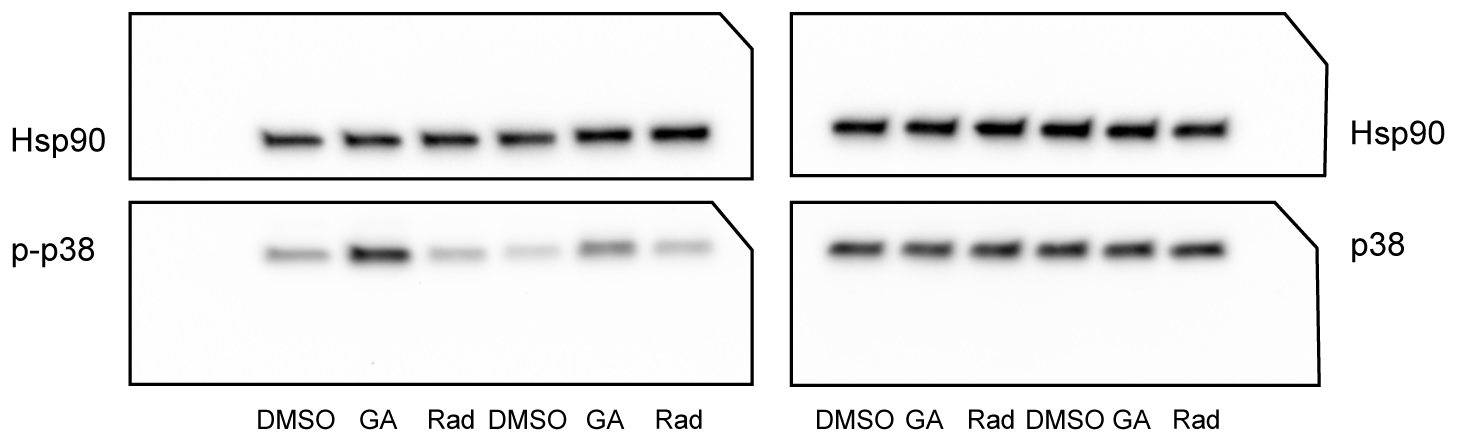

Supplement: S5 Fig — HEp-2 cells were serum-starved in HEPES-buffered medium before incubation with 10 μM GA or 1 μM radicicol (Rad) for 30 min. The cells were lysed and proteins were separated by SDS-PAGE. The membranes were cut above the molecular marker for 150 kDa and just below 75 kDa, 50 kDa and 25 kDa. Blots were probed with the indicated antibodies. Hsp90 was used as a loading control. (TIF) [file pone.0129214.s005.tif]

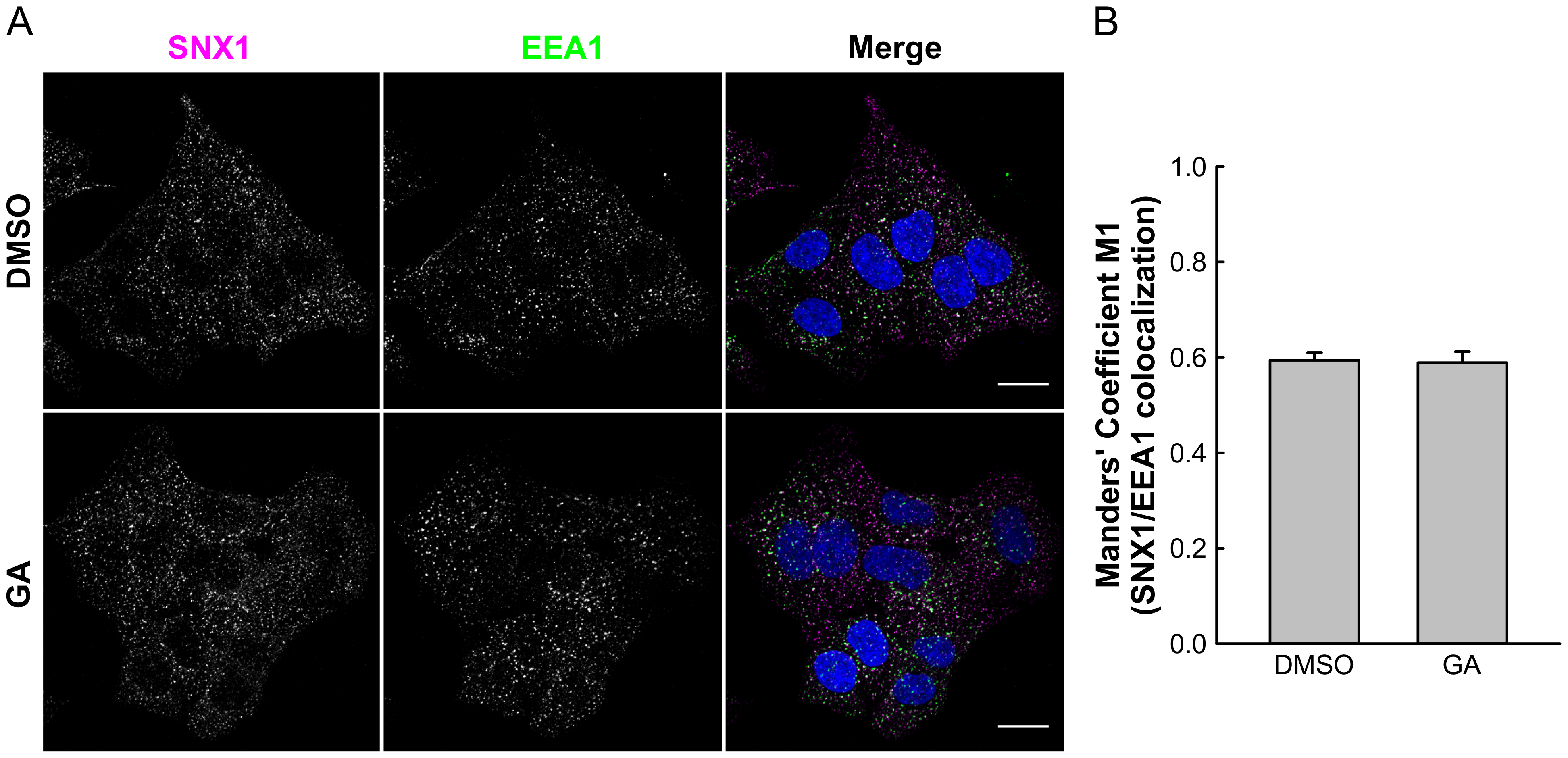

Supplement: S6 Fig — (A) HEp-2 cells were treated with 10 μM GA for 30 min and subsequently fixed, permeabilized and stained with antibodies against SNX1 (magenta) and EEA1 (green). DAPI is shown in blue. Scale bar 20 μm. (B) The colocalization between SNX1 and EEA1 was quantified using the coloc2 plugin in the Fiji software and is presented as the mean Manders’ colocalization coefficient for the ratio of SNX1 colocalizing with EEA1 + SEM. n = 3, with at least 59 cells quantified for each condition. * p ≤0.05, paired Student’s t-test. (TIF) [file pone.0129214.s006.tif]

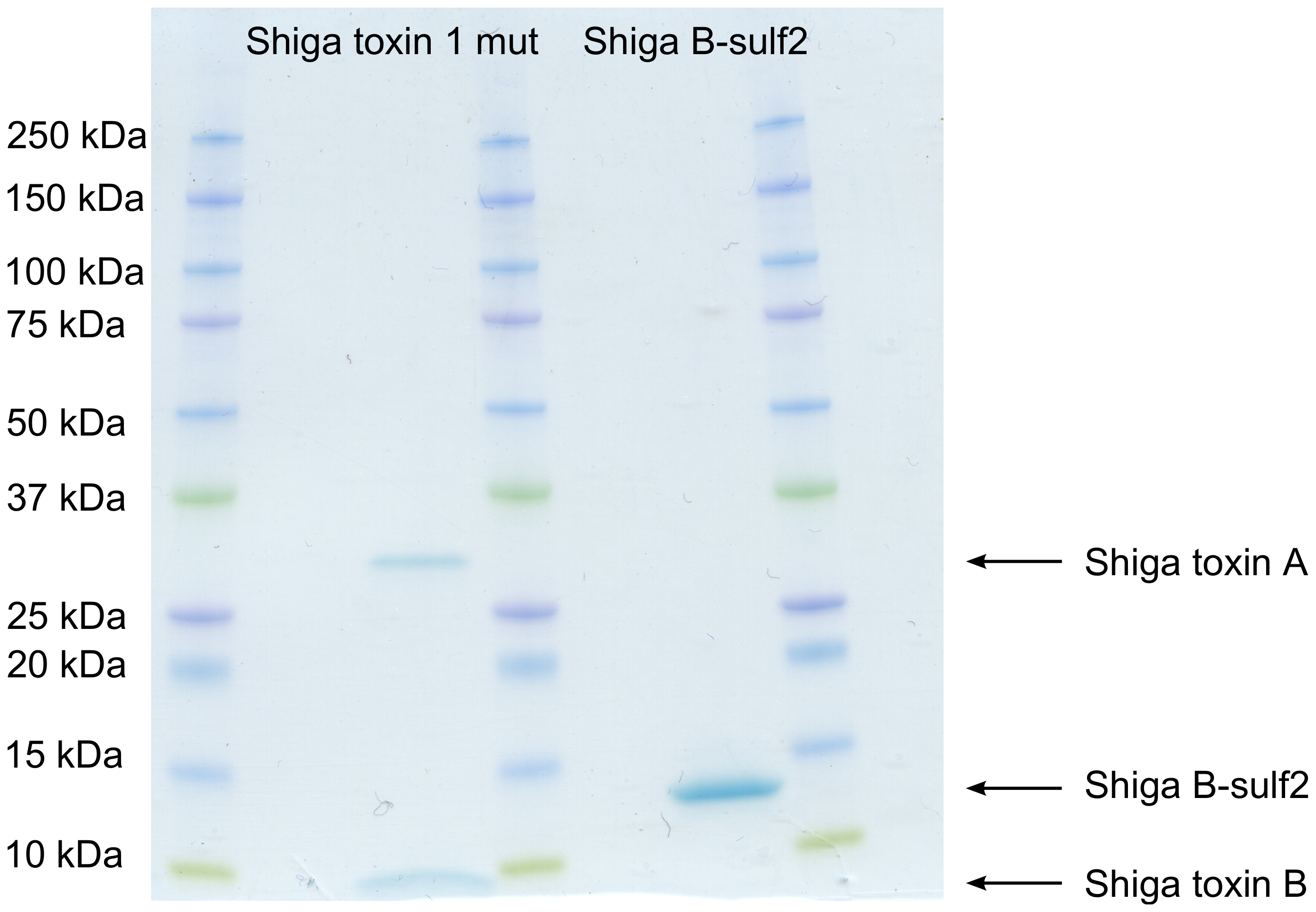

Supplement: S7 Fig — Coomassie-stained SDS-polyacrylamide gel (4–20%) showing a single band for Shiga B-sulf2. Purified Shiga toxin 1 mutant was included as a reference to show the A-moiety (~32 kDa) and B-subunits (~8 kDa) of Shiga toxin. Shiga B-sulf2 runs slightly higher than the B-subunit due to the additional sulfation sites. (TIF) [file pone.0129214.s007.tif]

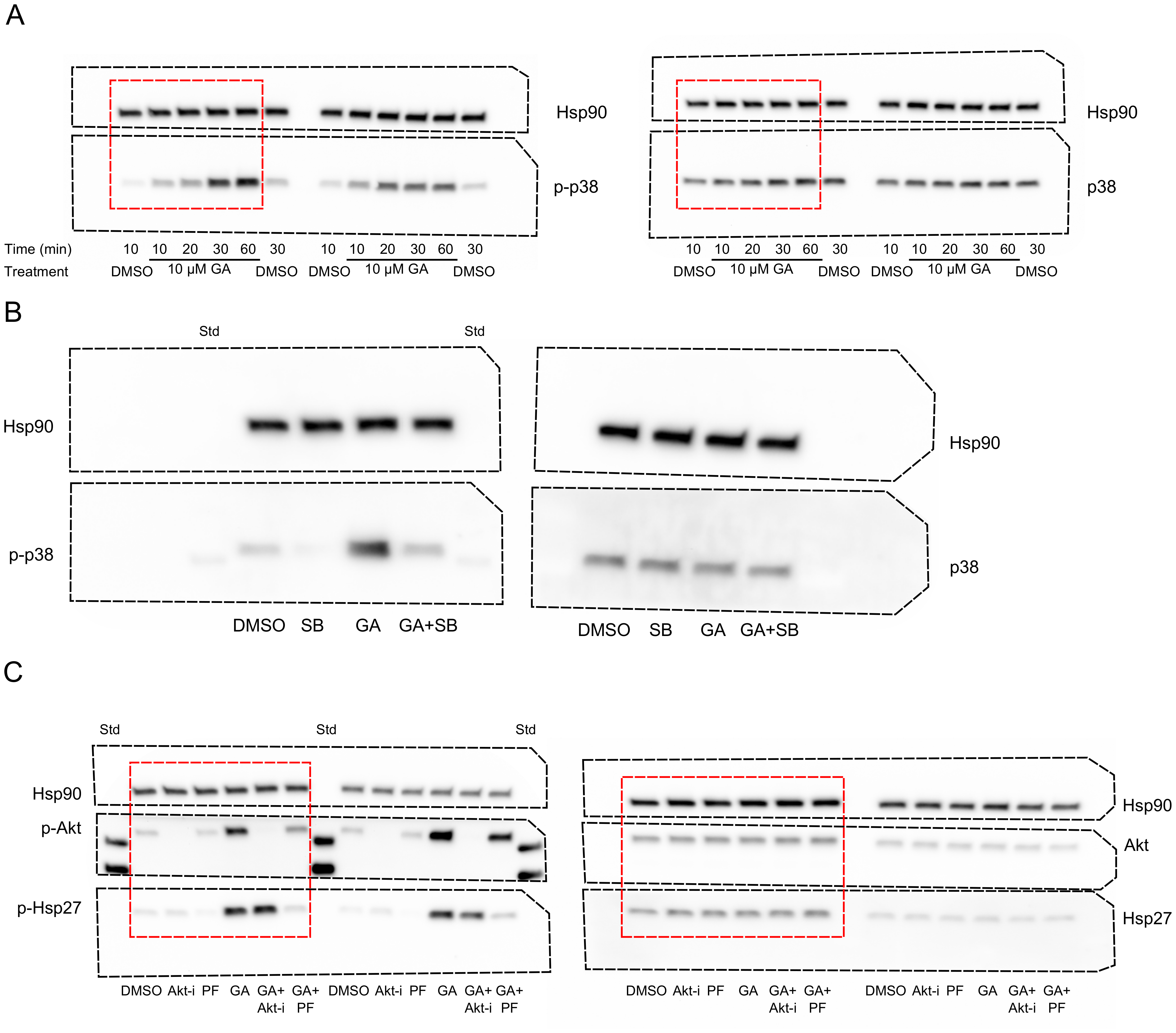

Supplement: S8 Fig — Original western blots for (A) Fig 6A, (B) Fig 6B, and (C) Fig 7A. The blots in A and B were cut between the molecular markers 150 and 250 kDa, 50 and 75 kDa, and 20 and 25 kDa. The blots in C were cut above 150 kDa and just below 75 kDa, 37 kDa and 15 kDa. The exposure time was optimized for each blot to give strong signals without saturation. The red boxes indicate the bands shown in Figs 6 and 7. (TIF) [file pone.0129214.s008.tif]
